# Supplementary material for: Trust in Institutions, Not in Political Leaders, Determines Compliance in COVID-19 Prevention Measures within Societies across the Globe
Source: Behav Sci (Basel). 2022 May 30;12(6):170. doi: 10.3390/bs12060170 (PMC9219766; doi:10.3390/bs12060170)
Supplement: Supplementary file 1 [file behavsci-12-00170-s001.zip › behavsci-1704830-supplementary.pdf]

Supplementary Information for

Trust in Institutions, Not in Political Leaders, Determines Compliance in COVID-19 Prevention Measures within Societies across the Globe

Ryan P. Badman [1,*], Ace X. Wang [2], Martin Skrodzki [3,4], Heng-chin Cho [5], David Aguilar-Lleyda [1], Naoko Shiono [1], Seng Bum Michael Yoo [6,7], Yen-Sheng Chiang [5] and Rei Akaishi [1]

[1] Center for Brain Science, RIKEN, Wako, Saitama 351-0198, Japan; david.aguilarlleyda@riken.jp (D.A.-L.); naoko.shiono@riken.jp (N.S.); rei.akaishi@riken.jp (R.A.)
[2] Economics Department, State University of New York at Binghamton, Binghamton, NY 13902, USA; xwang222@binghamton.edu
[3] RIKEN Interdisciplinary Theoretical and Mathematical Sciences Program, RIKEN, Wako, Saitama 351-0198, Japan; m.skrodzki@tudelft.nl
[4] Computer Graphics and Visualization, Dept. of InSy / EEMCS, TU Delft P.O. Box 5031, 2600 GA, Delft, The Netherlands
[5] Institute of Sociology, Academia Sinica, Taipei 11529, Taiwan; hengchin.cho@gmail.com (H.C.); chiangys@gate.sinica.edu.tw (Y.S.C.)
[6] Center for Neuroscience Imaging Research, Institute for Basic Science (IBS), Suwon 16419, Republic of Korea; sbyoo.ur.bcs@gmail.com
[7] Department of Biomedical Engineering, Sungkyunkwan University (SKKU), Suwon 16419, Republic of Korea
* Correspondence: ryan.badman113@gmail.com

This PDF file includes:

Figures S1 to S16

Tables S1 to S37

Other supplementary materials for this manuscript include the following:

Supplementary Data and Codes at the OSF directory https://osf.io/sevct/

SUPPLEMENTARY FIGURES AND TABLES

Summary of Contents:

Multiscale Structure of Government and Social Trust Across Countries/Territories, p. 2-7

Regression Variable Correlation Matrices, p. 7-9

Differences Between National Political Leader Trust and National Institutional Trust, p. 9-10

Government Trust versus Transparency Analysis (SEM), p. 10-13

Model Comparison for Trust versus Public Health Compliance and Beliefs, p. 13-20

Separate OLS Regressions for Each Individual Country/Territory (Analysis Check), p. 21-37

Public Health Compliance and Beliefs Per Country/Territory, p. 37

Standard Deviation Per Regression Variable, p. 37

Regression Checks on Transparency SEM Results, p. 38-41

Information Trust, and Public Health Compliance and Beliefs, p. 41-42

Community-related Trust, and Public Health Compliance and Beliefs (SEM and Regression), p. 42-44

General Trust (Yamagishi Scale), p. 44

National Identity, p. 44-45

Variance Inflation Factor Multicollinearity Test, p. 45

Demographics Per Country/Territory Summary, p. 46-52

Supplementary Information References, p. 53-54

<u>Multiscale Structure of Government and Social Trust Across Countries/Territories</u>

Overview: To prepare the main survey trust variables for analyses, we grouped them into the following categories based both on statistical and conceptual correlations: national institutional trust in public health institutions (Q73 and Q74, r=0.77), national political leader trust (Q19 and Q72, r=0.72), local institutional trust (Q55 and Q57, r = 0.48), local community trust (Q61 and Q62, r = 0.53), trust in strangers (Q58 and Q63, r = 0.45), trust in employers (Q64), social media trust (Q56), traditional news media trust (Q59), trust in science (Q97), and trust towards the World Health Organization (Q93).

The national government-related trust questions (national institutions and national political leaders) were phrased in the pandemic context by asking about absolute-scale trust: (1)

trust in Covid-19 related information from, and confidence in, national public health institutions (e.g. agencies), and (2) trust in Covid-19 related information from, and confidence in, the national political leaders. The trust question in each category was on a scale of "totally untrustworthy" to "very trustworthy", while the confidence question in each category was on a scale of "no confidence" to "high confidence". Thus, these two main categories of interest in our study were phrased and measured with directly comparable response scales.

The other broader trust questions were phrased in how the pandemic has altered one's trust in each category on a scale of "significantly decreased" to "significantly increased", and the scales were even so that some preference in one direction must be given by the subjects. The even-point scale of these questions was 1-10 (strong decrease to strong increase), thus we subtracted by 4.5 to set the midpoint to 0 in Fig. S1-S3. Despite the secondary trust questions being on a "change in trust" scale, it is important to note that our study is not longitudinal, thus these questions are measuring a composite of both current absolute trust levels (like the national government questions), plus perceptions of how trust has changed in those categories since the pandemic began. Thus, all trust questions in the survey are comparable and containing an absolute trust component, but future work would need a more structured longitudinal study to better understand the time-dependent trends in the trust variables.

Figures:

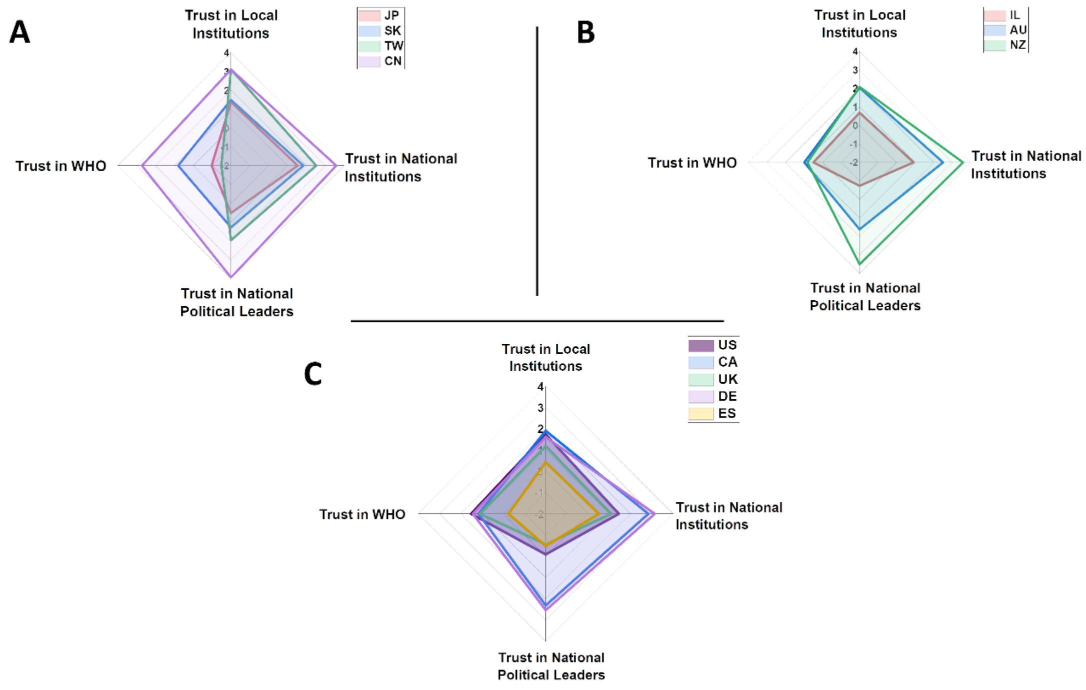

Figure S1: Baseline government-related trust across countries/territories
Radar plots of the mean values each category of trust used in the regression analyses, per country/territory. Standard ISO 3166 abbreviations for each country/territory are used in the legends. *Trust in National Institutions* is the mean of questions Q73 and Q74. Q73: Overall, how much do you trust the Covid-19-related information provided by your country's (territory's) government agency staff and bureaucrats, regardless of your distrust/trust of individual political leaders and heads of agencies? Q74: How much confidence do you have overall in the public

health-related bureaucratic agencies in your country (territory) (e.g. agencies for disease prevention and containment). *Trust in National Political Leaders* is the mean of Q19 and Q72. Q19: “Do you have confidence in your current NATIONAL government leaders' response to the Covid-19 pandemic?” Q72: “How much do you trust the Covid-19-related information provided by your country's (territory’s) political leaders? *Trust in Local Institutions* is a the mean of questions Q55 and Q57. Q55: “Did your trust in your local public officials decrease or increase during the Covid-19 pandemic?” Q57: “Did your trust in the safety of your local hospitals increase or decrease during the Covid-19 pandemic?” *Trust in the WHO* is just Q93: “Has the handling of the Covid-19 outbreak decreased or increased your trust in global institutions like the World Health Organization (WHO)?”.

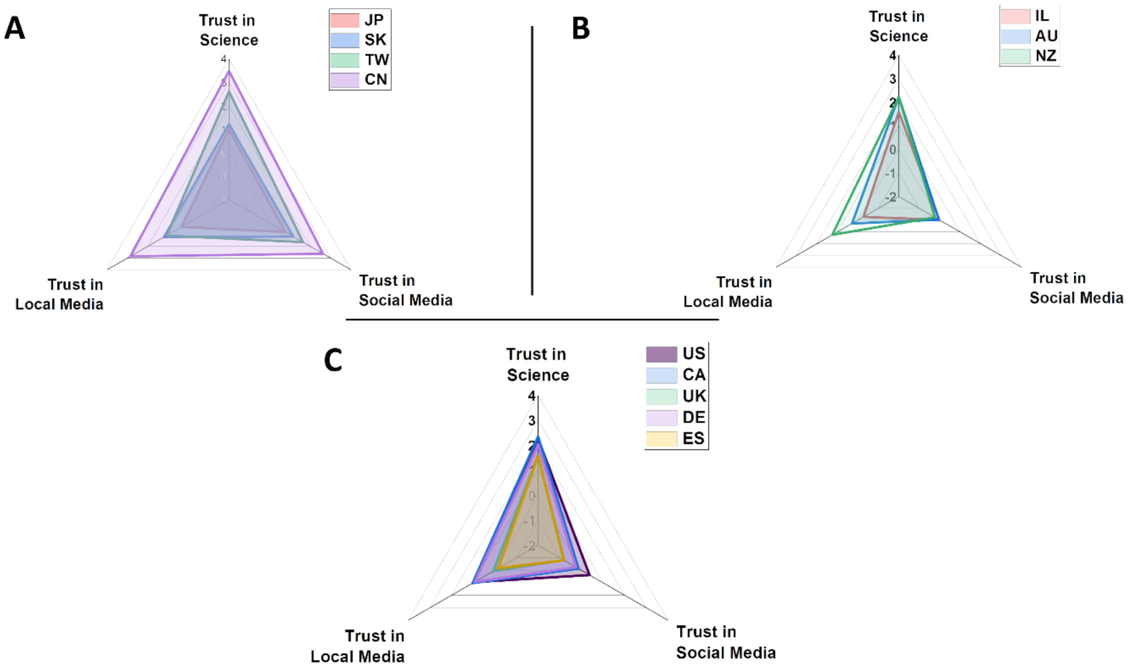

Figure S2: Baseline information-related trust across countries/territories
Radar plots of the mean values each category of information trust used in the regression analyses (*Trust in Social Media* in Q56, *Trust in Local Media* in Q59, and *Trust in Science* in Q97), per country/territory. Q56: “Has your trust in news and stories on social media (e.g. Facebook, Twitter, Instagram, Line, etc.) increased or decreased during the Covid-19 pandemic?” Q59: “Has your trust in local news media (local newspapers or TV channels) increased or decreased during the Covid-19 pandemic?” Q97: “Has the global handling of the Covid-19 outbreak increased or decreased your trust in scientific results generally?” Standard ISO 3166 abbreviations for each country/territory are used in the legends.

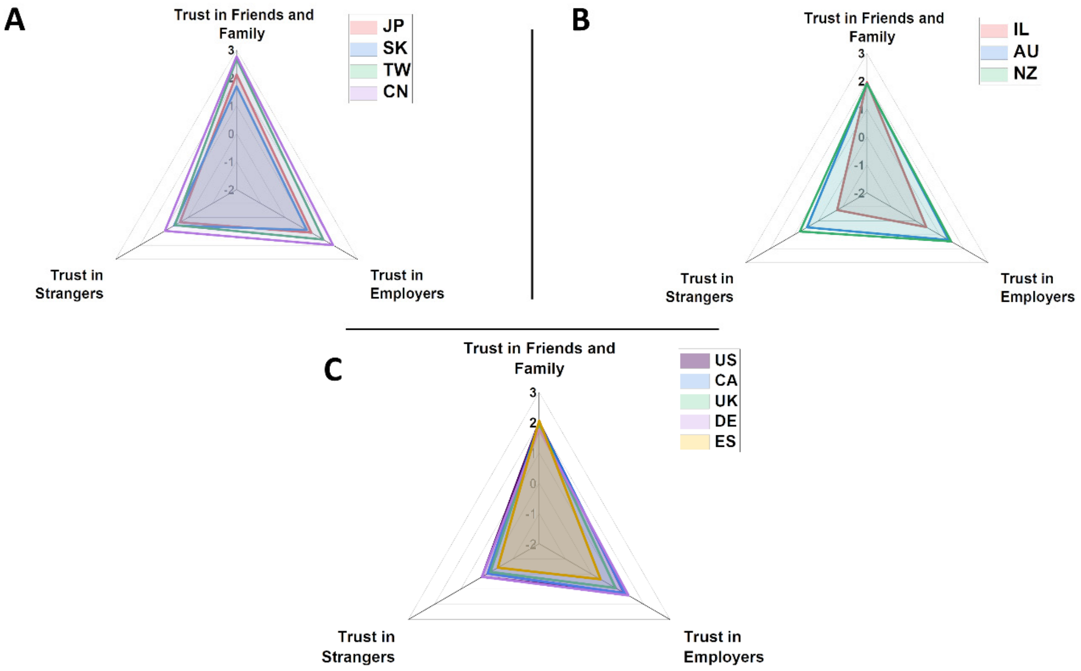

Figure S3: Baseline community-related trust across countries/territories
Radar plots of the mean values each category of community-related trust used in the regression analyses, per country/territory. *Trust in Friends and Family* is the mean of Q61 and Q62. Q61: "Overall, has your trust in your immediate family members decreased or increased during the Covid-19 pandemic?" Q62: "Overall, has your trust in your friends and social circle decreased or increased during the Covid-19 pandemic?" *Trust in Strangers* is the mean of Q58 and Q63. Q58: "Has your trust in being able to safely visit your local businesses (e.g.restaurants, bars, stores, etc.) increased or decreased during the Covid-19 pandemic?" Q63: "Overall, has your trust in acquaintances and strangers you encounter decreased or increased during the Covid-19 pandemic?" *Trust in Employers* is mean of Q64. Q64: "Overall, has your trust in your employers (or university administrators if student) decreased or increased during the Covid-19 pandemic? If you are unemployed/self-employed, answer instead for your perception of employers/administrators generally in your country/territory." Standard ISO 3166 abbreviations for each country/territory are used in the legends.

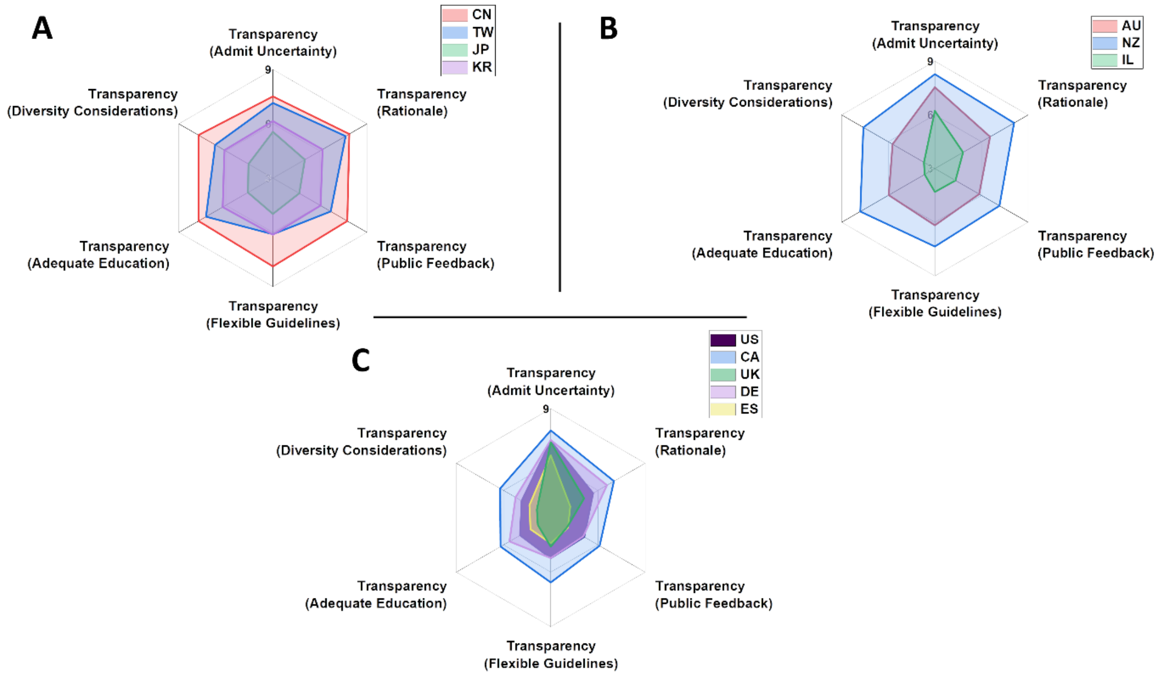

Figure S4: Transparency assessments across countries/territories
Radar plots of the mean response value per country/territory for each of the six transparency-related questions, paraphrased as follows. Q80: "Do officials make enough effort to explain their policies to the public?" (*Rationale*), Q81: "Do officials adequately consider public feedback when designing and implementing public health policies?" (*Public Feedback*). Q82: "Do the government officials provide Covid-19 health guidelines that are flexible enough to account for different contexts in your society" (*Flexible Guidelines*). Q83: "Do the government officials in charge of the pandemic response overall have enough training and education to provide public health advice" (*Adequate Education*), Q84: "How effective are government officials in communicating with members of the public who have diverse cultural values or political idealogies (*Diversity Considerations*). Q85: "Do you trust your public health officials more when they admit there is uncertainty in their predicitons or advice?" (*Admit Uncertainty*). Standard ISO 3166 abbreviations for each country/territory are used in the legends.

Summary comments about the multiscale per-country/territory trust plots

While local community trust was comparably high in all countries/territories studied, significant variation between countries/territories existed especially in institutional trust (national government), trust in science and the WHO, and trust in strangers. Outliers like the generally low trust Japan and generally high trust China agree with prior work (1, 2).

For China's high baseline trust results more specifically, authoritarianism or authoritarian tendencies of a government has been found to be an independent explanatory variable that significantly inflates institutional trust (especially trust in civic service) (3, 4). This trend may have been reproduced in our results of China, which has exceptionally high institutional trust among Asian cultures (Fig. S1). Some researchers have written off the well-documented high trust in China to authoritarian political propaganda campaigns, hierarchical cultures values (e.g.

Confucianism) and political fear while taking surveys (5). However, in the study of institutional trust in China by Yang & Tang they made the important point that the average citizen in any culture interacts with their institutions much more frequently than national politicians, and that many studies miss this critical institutional performance component when analyzing trust in governments in authoritarian countries (5). Work by Li also provides evidence that Chinese citizens in all demographics distinguish between different scales of government, trusting the national scale more than the local scale, and are sensitive to institutional performance during trust formation (2). Yang & Tang found that trust in administrative institutions is among the largest predictor for Chinese people's overall institutional trust, and that China's high institutional trust from the public is real (5). Additionally, considering the pandemic context of China's apparent low Covid-19 death count and competent pandemic management relative to many countries in the world (6, 7), public support domestically for the Chinese government in the pandemic context makes conceptual sense.

Last, for study limitations and possible future directions, the national government level trust generally across countries and territories could be investigated in greater detail than we have done here, to further understand the differences between institutional and political leader trust. For example, national institutions and political leader differ in number of significant ways: single individuals versus an aggregate of a large number of people, the rules and incentives governing the two types of government entities etc. In contrast to the individual character of the political leader, the public may have quite different trust levels for the individual heads of government agencies, versus the average staff within the agencies. The general methodology or policies that the institution follows (e.g. the scientific method) differ from the logic and incentives political leaders follow, and the trust towards these broader processes may differ as well (8, 9). Additionally, more detailed questions probing the differences between trust and/or confidence in competence ("assurance"-based trust (1)), versus more interpersonal trust in personal motivation, intentions and character, for the relevant agents in each trust category is also an important future direction, especially in cross-cultural comparisons (1, 10, 11).

Regression Variable Correlation Matrices

Table S1: Correlation matrix for major trust categories in the primary regression

Pairwise correlations

Variables	(1)	(2)	(3)	(4)	(5)	(6)	(7)	(8)
(1) Local Inst. Trust	1.000							
(2) Nat'l Inst. Trust	0.560	1.000						
(3) Nat'l Leader Trust	0.554	0.790	1.000					
(4) Trust in Science	0.466	0.491	0.423	1.000				
(5) Social Media Trust	0.453	0.313	0.372	0.284	1.000			
(6) News Media Trust	0.538	0.484	0.489	0.393	0.569	1.000		
(7) Community Trust	0.396	0.224	0.238	0.292	0.278	0.310	1.000	
(8) Trust in Strangers	0.410	0.237	0.321	0.178	0.364	0.390	0.354	1.000

Table S2: Correlation matrix for transparency questions

Pairwise correlations

Variables	(1)	(2)	(3)	(4)	(5)	(6)
(1) Q80	1.000					
(2) Q81	0.675	1.000				
(3) Q82	0.632	0.732	1.000			

(4) Q83	0.641	0.726	0.706	1.000		
(5) Q84	0.651	0.713	0.717	0.775	1.000	
(6) Q85	0.431	0.381	0.383	0.426	0.388	1.000

Table S3: Correlation matrix for demographics and a representative transparency question

Pairwise correlations

Variables	(1)	(2)	(3)	(4)	(5)	(6)	(7)	(8)	(9)	(10)	(11)	(12)	(13)
(1) Q80	1.000												
(2) Q18	-0.026	1.000											
(3) Q21	0.126	0.279	1.000										
(4) Q4	0.023	-0.067	0.051	1.000									
(5) Q5	-0.066	0.030	-0.074	-0.134	1.000								
(6) Q18	-0.026	1.000	0.279	-0.067	0.030	1.000							
(7) Q21	0.126	0.279	1.000	0.051	-0.074	0.279	1.000						
(8) Q22	0.146	-0.011	0.069	0.152	-0.050	-0.011	0.069	1.000					
(9) Q12	0.139	0.089	0.155	0.002	-0.177	0.089	0.155	0.134	1.000				
(10) Q13	-0.016	0.132	0.171	-0.087	-0.080	0.132	0.171	0.012	0.127	1.000			
(11) Q1	0.022	0.186	0.131	-0.003	0.119	0.186	0.131	0.010	0.046	-0.006	1.000		
(12) Q44	0.261	-0.011	0.056	0.147	-0.070	-0.011	0.056	0.092	0.088	-0.296	-0.038	1.000	
(13) Q8	0.123	0.052	0.077	0.234	-0.005	0.052	0.077	0.144	0.130	-0.215	0.127	0.206	1.000

Table S4: Correlation matrix for individual trust questions

Pairwise correlations

Variables	(1)	(2)	(3)	(4)	(5)	(6)	(7)	(8)	(9)	(10)	(11)	(12)	(13)	(14)	(15)
(1) Q19	1.000														
(2) Q72	0.724	1.000													
(3) Q73	0.666	0.830	1.000												
(4) Q74	0.571	0.700	0.767	1.000											
(5) Q55	0.507	0.490	0.490	0.471	1.000										
(6) Q57	0.390	0.377	0.398	0.450	0.479	1.000									
(7) Q61	0.189	0.154	0.158	0.157	0.253	0.315	1.000								
(8) Q62	0.214	0.214	0.212	0.212	0.296	0.329	0.527	1.000							
(9) Q58	0.250	0.229	0.199	0.164	0.313	0.349	0.179	0.275	1.000						
(10) Q63	0.275	0.264	0.223	0.173	0.288	0.247	0.221	0.394	0.452	1.000					
(11) Q64	0.300	0.316	0.300	0.282	0.331	0.347	0.289	0.374	0.328	0.402	1.000				
(12) Q56	0.356	0.334	0.301	0.287	0.437	0.341	0.235	0.251	0.295	0.328	0.272	1.000			
(13) Q59	0.434	0.476	0.468	0.441	0.513	0.411	0.259	0.284	0.351	0.312	0.339	0.569	1.000		
(14) Q93	0.253	0.348	0.342	0.381	0.299	0.255	0.105	0.183	0.163	0.176	0.249	0.240	0.388	1.000	
(15) Q97	0.371	0.416	0.449	0.475	0.388	0.414	0.229	0.282	0.157	0.146	0.281	0.284	0.393	0.440	1.0

Table S5: Correlation matrix for dependent variables, government trust questions, and transparency questions

Pairwise correlations

Variables	(1)	(2)	(3)	(4)	(5)	(6)	(7)	(8)	(9)	(10)	(11)	(12)	(13)	(14)	(15)
(1) Q32	1.000														
(2) Q34	0.396	1.000													
(3) Q42-Q43	0.148	0.175	1.000												
(4) Q19	-0.029	0.083	-0.070	1.000											
(5) Q72	0.025	0.131	0.019	0.724	1.000										
(6) Q73	0.047	0.146	0.075	0.666	0.830	1.000									
(7) Q74	0.088	0.188	0.114	0.571	0.700	0.767	1.000								
(8) Q55	0.018	0.122	-0.011	0.507	0.490	0.490	0.471	1.000							
(9) Q57	0.088	0.127	0.004	0.390	0.377	0.398	0.450	0.479	1.000						
(10) Q80	0.004	0.084	-0.012	0.620	0.606	0.619	0.551	0.489	0.382	1.000					
(11) Q81	-0.033	0.068	-0.049	0.635	0.612	0.604	0.542	0.568	0.403	0.675	1.000				
(12) Q82	-0.042	0.105	-0.014	0.598	0.601	0.575	0.525	0.539	0.395	0.632	0.732	1.000			
(13) Q83	-0.019	0.067	-0.051	0.647	0.639	0.643	0.569	0.542	0.420	0.641	0.726	0.706	1.000		
(14) Q84	-0.054	0.061	-0.073	0.655	0.627	0.599	0.531	0.562	0.415	0.651	0.713	0.717	0.775	1.000	
(15) Q85	0.031	0.163	0.122	0.339	0.411	0.479	0.463	0.303	0.283	0.431	0.381	0.383	0.426	0.388	1.000

Table S6: Correlation matrix for public health compliance and beliefs variables

	Mask-wearing	Social-Distancing	Perception of Benefit
Mask-wearing (Q32)	1.00		
Social-Distancing (Q34)	0.396	1.00	
Perception of Benefit (Q42-Q43)	0.148	0.175	1.00

<u>Differences Between National Political Leader Trust and National Institutional Trust</u>

To explore the differences in national political leader trust and national institutional trust within our data, we investigate how demographics, information trust, perceived degree of the national government listening to economic versus medical experts (Q75 and Q75-Q76) and one representative transparency question each independently impact both categories of national government trust. Fixed effects OLS with country/territory-level dummy variables are used as in the other OLS analyses.

Table S7: Linear regression summary factors predicting higher national political leader trust and higher national institutional trust

VARIABLES	(1)<br>National Political Leader Trust	(2)<br>National Institutional Trust
Transparency: Rationale	0.160***	0.214***
	(0.019)	(0.025)
Political Ideology (Conservative)	0.017	-0.082***
	(0.035)	(0.026)
Religiosity	0.018	-0.020*
	(0.020)	(0.011)
National Gov Listened to Medical Experts Enough	0.634***	0.572***
	(0.039)	(0.033)
How Much More National Gov Listens to Medical Experts Above Economic Experts	-0.152***	-0.177***
	(0.018)	(0.019)
Social Media Trust	0.026	-0.010
	(0.019)	(0.013)
Traditional Media Trust	0.090***	0.185***
	(0.017)	(0.023)
Gender (Female)	-0.008	-0.023
	(0.018)	(0.024)
Education Level	0.018	0.037**
	(0.011)	(0.013)
Medical Experience	0.005	0.004
	(0.016)	(0.013)
Income	-0.038***	-0.006
	(0.011)	(0.012)

Sufficient Safety Net	0.044***	0.018
	(0.011)	(0.014)
# of Household Minors	-0.001	-0.040**
	(0.015)	(0.013)
Urbanicity	0.000	0.014
	(0.012)	(0.014)
Experienced Pandemic Financial Hardship	-0.003	-0.034*
	(0.014)	(0.017)
Age Group	-0.011	-0.022*
	(0.013)	(0.012)
Constant	-0.183***	0.024
	(0.017)	(0.018)
Observations	3,328	3,328
R-squared	0.746	0.617

Notes: Standard errors in the parentheses are clustered at country/territory level
*p<0.10; **p<0.05; ***p<0.01

Government Trust versus Transparency Analysis (SEM)

A paraphrased reminder summary of the transparency questions follows:

- *Rationale:* "Do officials make enough effort to explain their policies to the public?" (Q80)
- *Public Feedback:* "Do officials adequately consider public feedback when designing and implementing public health policies?" (Q81)
- *Flexible Policies:* "Do the government officials provide Covid-19 health guidelines that are flexible enough to account for different contexts in your society" (Q82)
- *Officials Adequately Educated:* "Do the government officials in charge of the pandemic response overall have enough training and education to provide public health advice" (Q83)
- *Consider Diversity:* "How effective are government officials in communicating with members of the public who have diverse cultural values or political ideologies?" (Q84)
- *Preference for Uncertainty:* "Do you trust your public health officials more when they admit there is uncertainty in their predictions or advice?" (Q85)

Structural Equation Modeling

PDFs of the STATA model outputs for each model are provided in the Supplementary Data for goodness of fit, significance of pathways, etc.

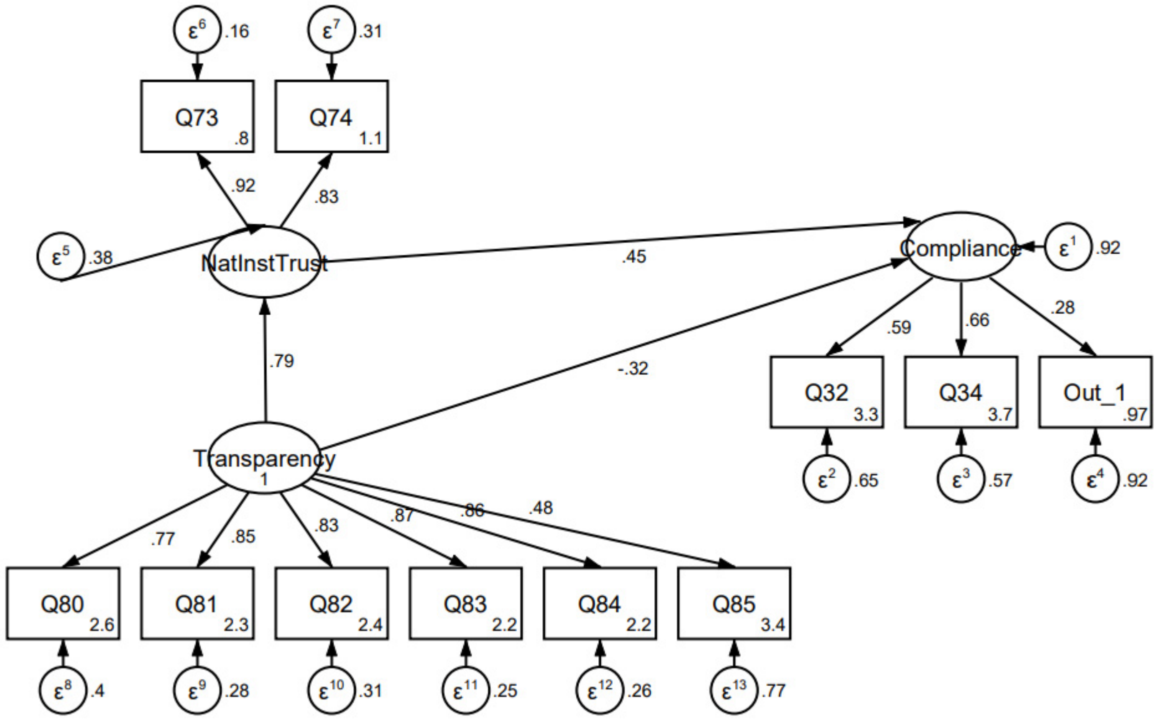

Figure S5: Structural equation modeling (SEM) for transparency, public health compliance & beliefs, and national institutional trust
Screenshot of SEM model built in STATA with standardized coefficients reported, for testing the relationship between transparency ("Transparency"), national institutional trust ("NatInstTrust"), and public health behaviors ("Compliance") (Fig. 3A). Question numbers match the survey questions, other than "Out_1" which is Q42-Q43. Standardized coefficients are reported above each pathway. RMSEA for this model is 0.074. Additional model information from STATA output is provided in Supplementary Appendix 3.

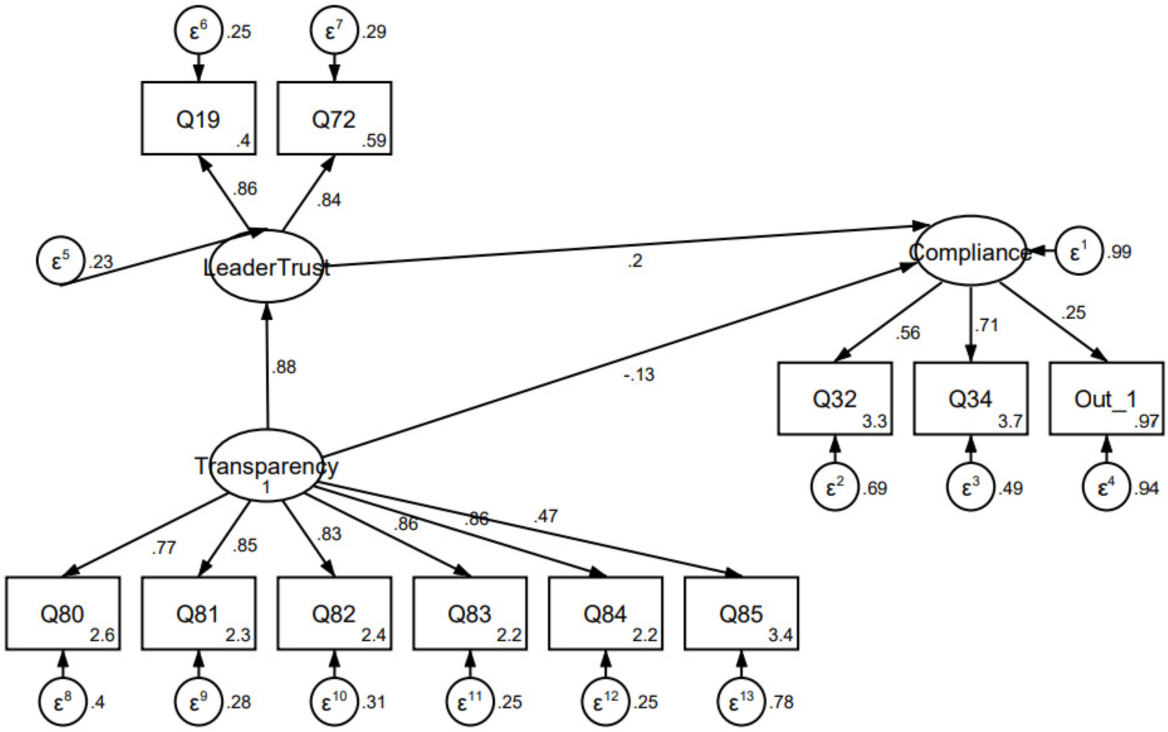

Figure S6: Structural equation modeling (SEM) for transparency, public health compliance & beliefs, and national political leader trust

Screenshot of SEM model built in STATA with standardized coefficients reported, for testing the relationship between transparency (“Transparency”), national political leader trust (“LeaderTrust”), and public health behaviors (“Compliance”) (Fig. 3B). Question numbers match the survey questions, other than “Out_1” which is Q42-Q43. Standardized coefficients are reported above each pathway. RMSEA for this model is 0.064. Additional model information from STATA output is provided in Supplementary Appendix 3.

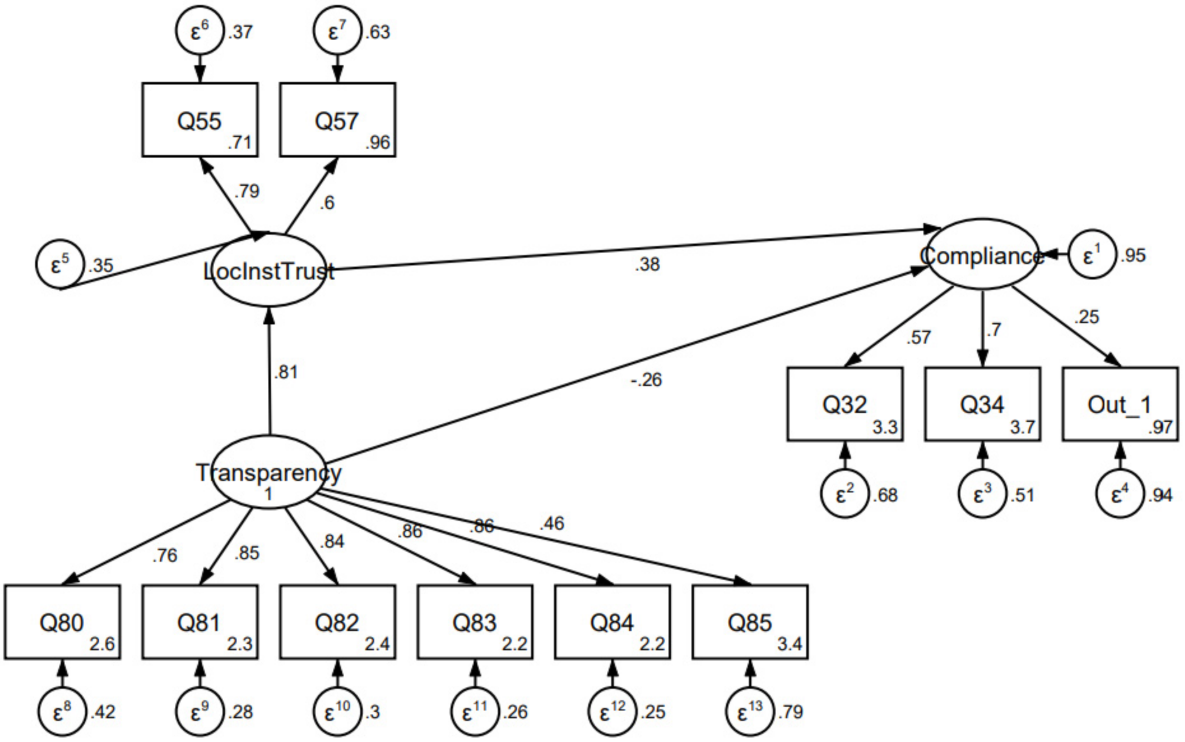

Figure S7: Structural equation modeling (SEM) for transparency, public health compliance & beliefs, and local institutional trust
Screenshot of SEM model built in STATA with standardized coefficients reported, for testing the relationship between transparency ("Transparency"), local institutional trust ("LocInstTrust"), and public health behaviors ("Compliance") (Fig. 3C). Question numbers match the survey questions, other than "Out_1" which is Q42-Q43. Standardized coefficients are reported above each pathway. RMSEA for this model is 0.058. Additional model information from STATA output is provided in Supplementary Appendix 3.

Model Comparison for Trust versus Public Health Compliance and Beliefs

Overview

Fixed effects OLS is the gold standard in socioeconometric analysis (12). Still, to ensure the robustness of our analysis results of the different effects of national institutional trust versus national political leader trust on public health compliance and beliefs in the effectiveness of public health measures, we compared multiple fixed and mixed effect regression models using a reduced set of the most impactful variables from the main Table 1 regression. We tested the following linear models: fixed country/territory effects OLS regression, a random intercept model with country/territory-level intercepts, and a random slope model with random slopes on national institutional trust and national political leader trust (the main variables of interest in our study). These models were chosen in terms of stepwise increases in complexity (13).

Brief Summary of Results

We find strong agreement between global coefficients in the full-variable-list fixed effects OLS regression in Table 1, the reduced-variable-list fixed effects OLS below (Table S8), and the random intercept model (Table S9). Additionally, the marginal effects per country/territory agree well between the fixed effects model and the random intercept model (Fig. 3, Fig. S10), solidifying robustness of our analysis results.

Furthermore, the fixed effects OLS models had lower AIC than the random intercept model, but higher BIC than the random intercept model. Given the strong agreement between global coefficients and marginal effects between the two models, and the lower AIC, we conclude our choice of fixed effects OLS in the full regression Table 1 was justified. Additionally, overall the fixed effects OLS had more conservative estimates of regression coefficient magnitudes and statistical significance than the random intercept model, so we have presented the more conservative results in the main text.

Last, during the exploratory effort to investigate random slope comparison, the two random slopes model threw a "singular boundary error" from having near zero random slope values, a common sign of over-fitting and over-parametrization, leading us to reject this model. Further diagnostic efforts of using just one random slope for national political leader trust found this simplest one slope model could not converge. Generally, random slopes models require at least ~25 countries/territories (with ~50 or more subjects per country/territory) for trustworthy fitting, so random slopes were not expected to perform well given the constraints of our data (14).

Equations:

Fixed effects OLS is summarized in the main text Methods.

Random Intercept Model:
Level 1: Yij=β0j+β1j (Nat. Inst. Trust) +β2j (Nat. Political Leader Trust)+etc……+Rij
Level 2: β0j=γ00+U0j

Random Slope Model:
Level 1: Yij=β0j+β1j(Nat. Inst. Trust)+β2j(Nat. Political Leader Trust)+etc……+Rij
Level 2: β0j=γ00+U0j
Level 2: β1j=γ01+U0j
Level 2: β2j=γ02+U0j

Results
The regression tables are as follows:

Table S8: Reduced Variable List, Fixed Effects OLS

VARIABLES	(1)<br>Mask-wearing	(2)<br>Social-Distancing	(3)<br>Belief in Effectiveness of Measures
Trust in Science	0.021***	0.022***	0.025***
	(0.005)	(0.005)	(0.006)
National Institutional Trust	0.033***	0.034***	0.060***
	(0.008)	(0.007)	(0.009)

National Political Leader Trust	-0.015*	-0.007	-0.023***
	(0.008)	(0.007)	(0.009)
National Institutional Trust # National Political Leader Trust Interaction Term (test interaction term between national institutional trust and political leader trust)	0.013***	0.015***	0.010**
	(0.004)	(0.004)	(0.005)
Local Institutional Trust	0.016***	0.020***	0.024***
	(0.006)	(0.005)	(0.006)
Trust in Strangers	-0.044***	-0.027***	-0.074***
	(0.005)	(0.004)	(0.005)
Local Community Trust	0.014***	0.016***	0.010*
	(0.005)	(0.004)	(0.005)
Gender (Female)	0.023***	0.038***	0.021**
	(0.009)	(0.007)	(0.009)
Education Level	0.008*	0.004	0.009*
	(0.004)	(0.004)	(0.005)
Income	-0.001	-0.010**	0.010*
	(0.005)	(0.004)	(0.005)
Sufficient Safety Net	0.017***	0.005	0.009*
	(0.005)	(0.004)	(0.005)
Political Ideology (Conservative)	-0.014***	-0.002	-0.022***
	(0.005)	(0.004)	(0.005)
Age Group	0.007	0.020***	-0.007
	(0.005)	(0.004)	(0.005)
CA	-0.064***	-0.010	0.043*
	(0.021)	(0.018)	(0.023)
UK	-0.057***	-0.011	0.002
	(0.020)	(0.017)	(0.022)
DE	0.054***	-0.048***	-0.047**
	(0.021)	(0.018)	(0.023)
ES	0.012	-0.001	0.053**
	(0.021)	(0.018)	(0.023)
IL	-0.038*	-0.110***	0.026
	(0.021)	(0.018)	(0.023)
AU	-0.178***	-0.027	-0.035
	(0.021)	(0.017)	(0.022)
NZ	-0.253***	-0.019	-0.054**
	(0.024)	(0.020)	(0.025)
JP	0.125***	-0.034**	-0.052**
	(0.020)	(0.017)	(0.022)
KR	-0.090***	-0.143***	-0.142***

	(0.020)	(0.017)	(0.022)
TW	0.021	-0.151***	-0.245***
	(0.021)	(0.018)	(0.023)
CN	-0.077***	-0.085***	-0.220***
	(0.020)	(0.017)	(0.022)
Observations	3,113	3,155	3,240
R-squared	0.174	0.138	0.219

Standard errors are given in parentheses and are unclustered for model comparison
*p<0.10; **p<0.05; ***p<0.01

Table S9: Reduced Variable List, Random Intercept Model

VARIABLES	(1) Mask-Wearing	(2) Social-Distancing	(3) Belief in Effectiveness of Measures
Trust in Science	0.020***	0.022***	0.025***
	(0.005)	(0.005)	(0.006)
National Institutional Trust	0.033***	0.034***	0.060***
	(0.008)	(0.007)	(0.009)
National Political Leader Trust	-0.016**	-0.007	-0.024***
	(0.008)	(0.007)	(0.009)
National Institutional Trust # National Political Leader Trust Interaction Term (test interaction term between national institutional trust and political leader trust)	0.012***	0.015***	0.010**
	(0.004)	(0.004)	(0.005)
Local Institutional Trust	0.016***	0.019***	0.024***
	(0.006)	(0.005)	(0.006)
Trust in Strangers	-0.044***	-0.027***	-0.074***
	(0.005)	(0.004)	(0.005)
Local Community Trust	0.014***	0.016***	0.010*
	(0.005)	(0.004)	(0.005)
Gender (Female)	0.023***	0.037***	0.021**
	(0.009)	(0.007)	(0.009)
Education Level	0.009*	0.004	0.009*
	(0.004)	(0.004)	(0.005)
Income	-0.001	-0.010**	0.010*
	(0.005)	(0.004)	(0.005)
Sufficient Safety Net	0.017***	0.005	0.009*
	(0.005)	(0.004)	(0.005)
Political Ideology (Conservative)	-0.014***	-0.002	-0.022***
	(0.005)	(0.004)	(0.005)
Age Group	0.007	0.020***	-0.007
	(0.005)	(0.004)	(0.005)
Observations	3,113	3,155	3,240

Number of groups	12	12	12

Standard errors are given in parentheses and are unclustered for model comparison
*p<0.10; **p<0.05; ***p<0.01

Error Message: Reduced variable list, random slope model, one random slope on national political leader trust: Taken from the R script in Supplementary Data, the error message given when trying to run a more complex multilevel random slope model (two slopes).

For mask-wearing as output variable:

```
" trust_model1 <- lmer(q32_maskPercent ~ index_gender + q1_age + q4_edu + q44_SafetyNet +
q8_inc
              + q18_politic +   + index_local_community_trust
              + index_local_institutional_trust
              + index_national_institutional_trust
              + index_national_political_leader_trust
              + index_trust_science
              + index_trust_strangers + (1 +
index_national_political_leader_trust|country)
                +
index_national_institutional_trust:index_national_political_leader_trust
              + (1|country) ,
              data = survey_data,
              REML = FALSE)


In checkConv(attr(opt, "derivs"), opt$par, ctrl = control$checkConv,  :

  Model failed to converge with max|grad| = 0.0116711 (tol = 0.002, component 1)
```

For social-distancing as output variable:

```
"Warning messages:

1: In checkConv(attr(opt, "derivs"), opt$par, ctrl = control$checkConv,  :

  unable to evaluate scaled gradient

2: In checkConv(attr(opt, "derivs"), opt$par, ctrl = control$checkConv,  :

  Model failed to converge: degenerate  Hessian with 1 negative eigenvalues

""' "
```

Error Message: reduced variable list, two random slope model, one random slope on national political leader trust and one random slope on national institutional trust: Taken

from the R script in Supplementary Data, the error message given when trying to run a more complex multilevel random slope model (one slopes).

```
" trust_model1 <- lmer(q32_maskPercent ~ index_gender + q1_age + q4_edu + q44_SafetyNet + q8_inc
                + q18_politic +   + index_local_community_trust
                + index_local_institutional_trust
                + index_national_institutional_trust
                + index_national_political_leader_trust
                + index_trust_science
                + index_trust_strangers + (1 + index_national_political_leader_trust|country) +

(1 + index_national_institutional_trust|country) + index_national_institutional_trust:index_national_political_leader_trust
                + (1|country) ,
                data = survey_data,
                REML = FALSE)


"boundary (singular) fit: see ?isSingular"
```

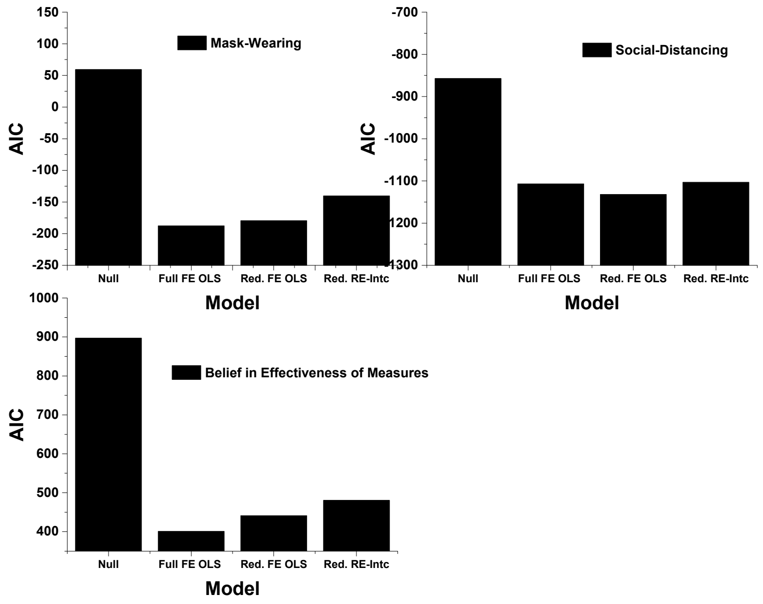

Figure S8: Akaike information criterion (AIC) model comparison
Comparison of AIC values for the null random intercept model with country/territory-level intercepts (*Null*), the full variable list fixed effects (FE) ordinary least squares (OLS) linear regression model (*Full FE OLS*, Table 1), the reduced variable list FE OLS (*Red. FE OLS)*, and the reduced variable list random intercept model (*Red. RE-Intc).*

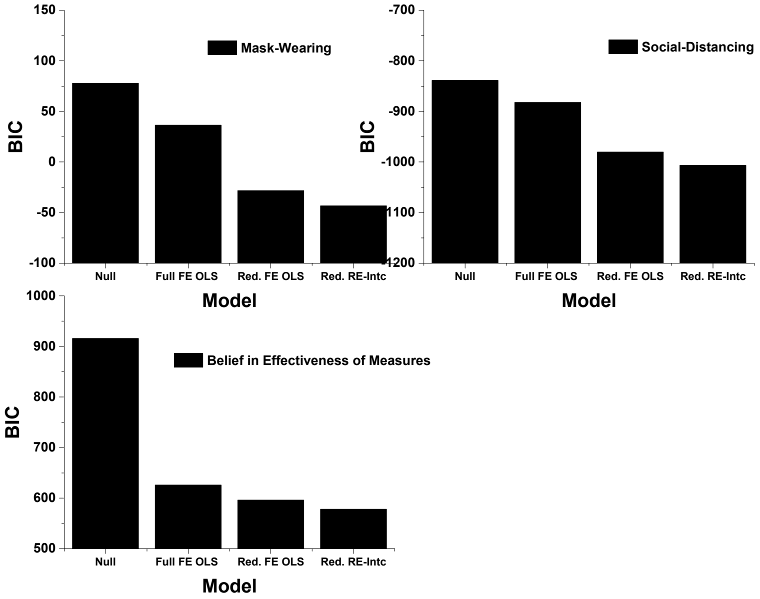

Figure S9: Bayesian information criterion (BIC) model comparison
Comparison of BIC values for the null random intercept model with country/territory-level intercepts (*Null*), the full variable list fixed effects (FE) ordinary least squares (OLS) linear regression model (*Full FE OLS*, Table 1), the reduced variable list FE OLS (*Red. FE OLS)*, and the reduced variable list random intercept model (*Red. RE-Intc).*

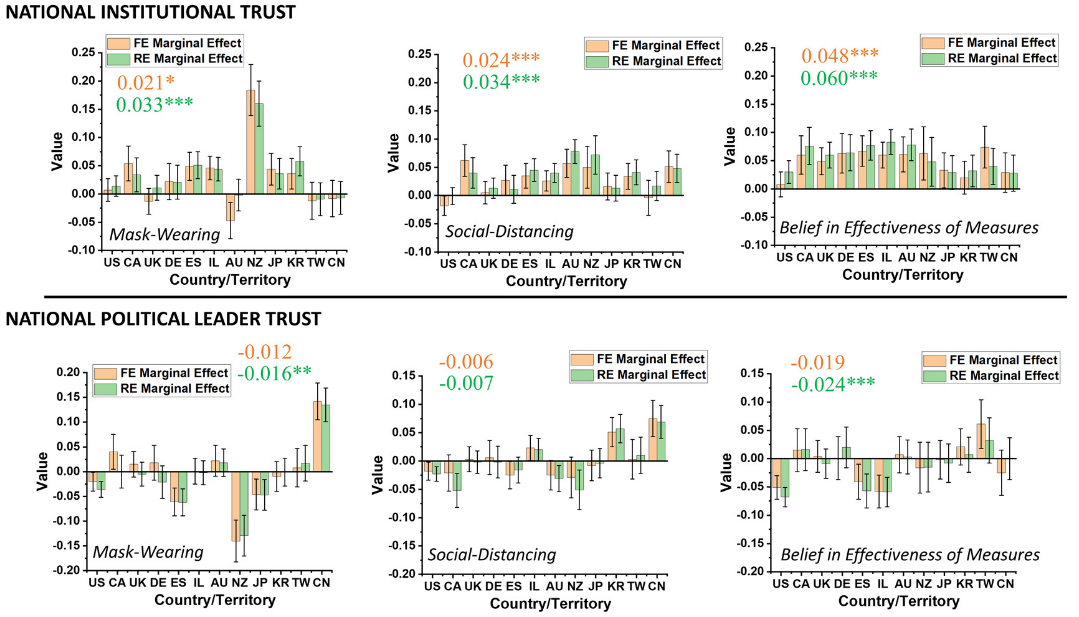

Figure S10: Marginal effects comparison between fixed effects OLS and random intercept models

Comparison of marginal effects per country/territory for national institutional trust (top) and national political leader trust (bottom), between the lowest AIC model of the full variable list linear regression model (*Full FE OLS*, Table 1, Fig. 3) (orange), and the lowest BIC reduced variable list random intercept model (*Red. RE-Intc*, Table S9) (green). The ICC values for the reduced-variable-list random intercept model were 0.142, 0.060, and 0.113 for mask-wearing, social-distancing and belief in effectiveness of measures respectively. Overall, the marginal effects results are highly consistent between the two modeling approaches. The global regression coefficients and their significance are reported for each model and variable in colors that match the bar colors (*p<0.10; **p<0.05; ***p<0.01). Error bars are standard errors of the mean.

Separate OLS Regressions for Each Individual Country/Territory (Analysis Check)

As a further robustness check, we ran the Table 1 regression variable list for twelve separate single country/territory OLS regressions (14). As shown in Fig. S11 and Tables S10-21, the marginal effects results from Fig. 3 agree well with simpler individual country/territory analysis' regression coefficients.

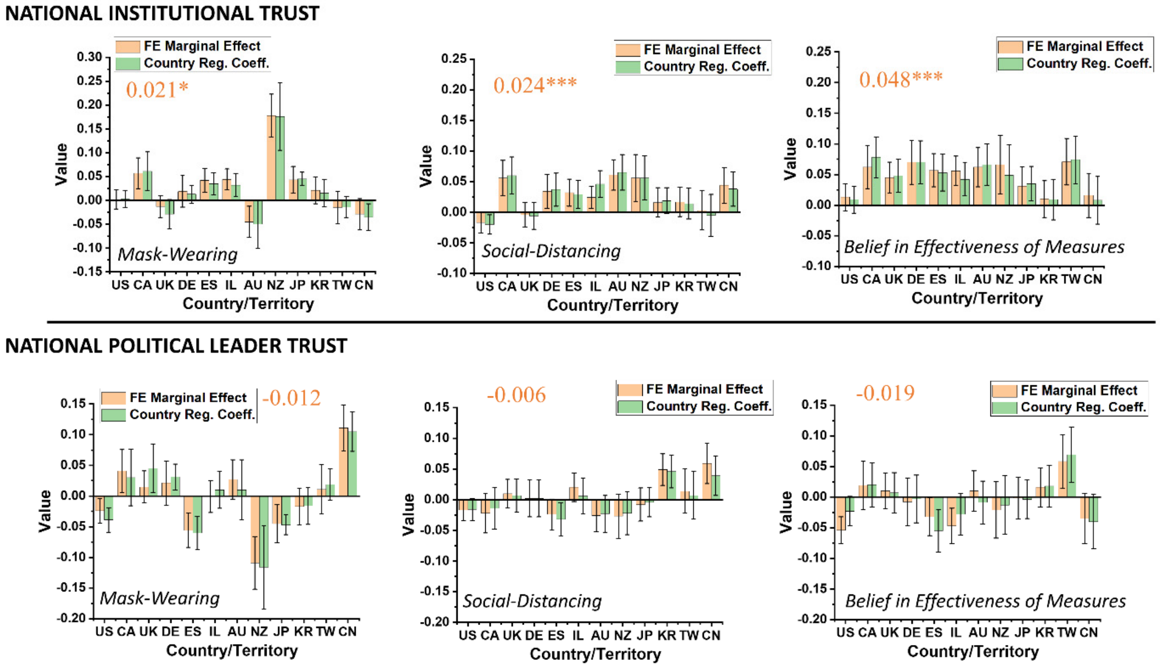

Figure S11: Single country/territory regressor coefficients comparison
Comparison of marginal effects per country/territory for national institutional trust and national political leader trust for the full variable list linear regression model (*Full FE OLS*, Table 1, Fig. 3), and the single country/territory OLS regression coefficients for these two variables per country/territory (below, Table S10-21). The global regression coefficients and their significance are reported for each model from Table 1 in orange text (*p<0.10; **p<0.05; ***p<0.01). Error bars are standard errors of the mean.

Table S10: US Independent OLS Results

VARIABLES	(1) Mask-wearing	(2) Social-Distancing	(3) Belief in Effectiveness of Measures
Trust in WHO	0.010	0.014	-0.009
	(0.016)	(0.015)	(0.020)
Trust in Science	0.032**	0.043***	0.064***
	(0.015)	(0.014)	(0.018)

National Institutional Trust	0.003	-0.020	0.009
	(0.018)	(0.016)	(0.022)
National Political Leader Trust	-0.039**	-0.016	-0.023
	(0.020)	(0.018)	(0.024)
Local Institutional Trust	0.006	0.003	0.027
	(0.015)	(0.014)	(0.019)
Trust in Strangers	-0.049***	-0.020	-0.071***
	(0.014)	(0.013)	(0.018)
Trust in Employers	0.007	0.022*	0.018
	(0.013)	(0.012)	(0.016)
Local Community Trust	-0.011	0.015	0.026
	(0.014)	(0.013)	(0.017)
Social Media Trust	-0.018	-0.029**	-0.033*
	(0.016)	(0.014)	(0.020)
Traditional Media Trust	-0.001	0.001	0.010
	(0.016)	(0.015)	(0.020)
General Trust (Local)	0.030	0.017	-0.014
	(0.018)	(0.017)	(0.023)
General Trust (Global)	-0.003	-0.025	-0.016
	(0.018)	(0.016)	(0.022)
Gender (Female)	0.008	0.004	0.019
	(0.025)	(0.023)	(0.031)
Education Level	0.002	-0.015	-0.002
	(0.013)	(0.012)	(0.016)
Medical Experience	0.000	0.002	-0.010
	(0.012)	(0.011)	(0.015)
Income	-0.013	-0.019	0.021
	(0.016)	(0.015)	(0.020)
Sufficient Safety Net	0.031**	0.008	-0.027
	(0.015)	(0.013)	(0.018)
# of Household Minors	-0.003	0.017	-0.019
	(0.014)	(0.012)	(0.017)
Political Ideology (Conservative)	0.003	-0.005	-0.031**
	(0.013)	(0.012)	(0.016)
Religiosity	-0.004	0.011	-0.019
	(0.013)	(0.012)	(0.016)
Urbanicity	0.017	-0.021*	0.025
	(0.012)	(0.011)	(0.015)
Experienced Pandemic Financial Hardship	0.007	-0.002	-0.046***
	(0.012)	(0.011)	(0.015)
Age Group	0.016	0.021*	-0.014
	(0.013)	(0.012)	(0.017)
National Identity 1	0.023	0.029*	0.024
	(0.018)	(0.017)	(0.023)
National Identity 2	-0.001	-0.014	0.010
	(0.019)	(0.017)	(0.024)
Observations	326	327	330

R-squared	0.156	0.168	0.305

Standard errors are given in parenthesis
*p<0.10; **p<0.05; ***p<0.01

Table S11: CA Independent OLS Results

VARIABLES	(1) Mask-wearing	(2) Social-Distancing	(3) Belief in Effectiveness of Measures
Trust in WHO	-0.034	0.004	-0.005
	(0.026)	(0.018)	(0.020)
Trust in Science	0.016	0.011	0.021
	(0.024)	(0.018)	(0.019)
National Institutional Trust	0.061	0.060**	0.078**
	(0.041)	(0.030)	(0.033)
National Political Leader Trust	0.030	-0.014	0.020
	(0.046)	(0.034)	(0.036)
Local Institutional Trust	-0.041	-0.002	-0.000
	(0.030)	(0.021)	(0.023)
Trust in Strangers	-0.012	-0.040**	-0.089***
	(0.026)	(0.018)	(0.021)
Trust in Employers	0.003	-0.001	0.037**
	(0.022)	(0.015)	(0.017)
Local Community Trust	0.045*	0.051***	-0.011
	(0.024)	(0.017)	(0.019)
Social Media Trust	-0.014	-0.006	0.009
	(0.023)	(0.017)	(0.019)
Traditional Media Trust	-0.042	-0.063***	0.032
	(0.030)	(0.021)	(0.024)
General Trust (Local)	0.003	0.024	0.013
	(0.032)	(0.022)	(0.024)
General Trust (Global)	-0.001	0.013	-0.039*
	(0.030)	(0.021)	(0.023)
Gender (Female)	-0.030	0.006	-0.020
	(0.041)	(0.028)	(0.032)
Education Level	-0.026	-0.018	0.021
	(0.023)	(0.016)	(0.018)
Medical Experience	0.005	-0.002	-0.023
	(0.020)	(0.014)	(0.016)
Income	0.004	-0.001	0.033*
	(0.024)	(0.017)	(0.019)
Sufficient Safety Net	0.005	-0.020	0.002
	(0.023)	(0.016)	(0.018)
# of Household Minors	0.005	0.016	0.023
	(0.028)	(0.018)	(0.021)
Political Ideology (Conservative)	-0.052**	0.008	-0.008
	(0.023)	(0.016)	(0.018)

Religiosity	0.010	-0.016	0.015
	(0.021)	(0.015)	(0.017)
Urbanicity	0.004	-0.009	-0.007
	(0.022)	(0.016)	(0.018)
Experienced Pandemic Financial Hardship	-0.019	-0.005	-0.014
	(0.022)	(0.016)	(0.018)
Age Group	-0.017	0.011	-0.036**
	(0.022)	(0.015)	(0.017)
National Identity 1	-0.029	-0.003	0.031
	(0.034)	(0.025)	(0.027)
National Identity 2	0.008	-0.015	0.007
	(0.034)	(0.026)	(0.027)
Observations	243	238	248
R-squared	0.120	0.134	0.331

Standard errors are given in parenthesis
*p<0.10; **p<0.05; ***p<0.01

Table S12: UK Independent OLS Results

VARIABLES	(1) Mask-wearing	(2) Social-Distancing	(3) Belief in Effectiveness of Measures
Trust in WHO	0.016	-0.015	-0.018
	(0.028)	(0.019)	(0.024)
Trust in Science	0.025	0.030*	0.048**
	(0.026)	(0.018)	(0.022)
National Institutional Trust	-0.029	-0.006	0.048*
	(0.031)	(0.022)	(0.027)
National Political Leader Trust	0.045	0.007	0.007
	(0.039)	(0.027)	(0.033)
Local Institutional Trust	0.017	-0.016	0.000
	(0.025)	(0.018)	(0.022)
Trust in Strangers	-0.085***	-0.029*	-0.065***
	(0.025)	(0.017)	(0.021)
Trust in Employers	-0.014	0.022	-0.023
	(0.020)	(0.014)	(0.017)
Local Community Trust	0.028	0.007	0.019
	(0.022)	(0.015)	(0.018)
Social Media Trust	-0.020	0.015	-0.041*
	(0.026)	(0.019)	(0.023)
Traditional Media Trust	0.032	0.011	0.024
	(0.026)	(0.018)	(0.022)
General Trust (Local)	0.002	0.028	0.011
	(0.029)	(0.021)	(0.025)
General Trust (Global)	-0.015	-0.009	-0.005
	(0.034)	(0.023)	(0.029)
Gender (Female)	0.086**	0.081***	0.013

	(0.041)	(0.028)	(0.035)
Education Level	0.020	0.023*	-0.020
	(0.018)	(0.013)	(0.016)
Medical Experience	-0.021	0.005	-0.035*
	(0.024)	(0.016)	(0.020)
Income	-0.031	-0.022	0.043
	(0.033)	(0.023)	(0.028)
Sufficient Safety Net	-0.021	0.001	-0.001
	(0.021)	(0.014)	(0.018)
# of Household Minors	-0.010	0.016	-0.037**
	(0.021)	(0.015)	(0.018)
Political Ideology (Conservative)	-0.037	0.013	-0.019
	(0.027)	(0.019)	(0.023)
Religiosity	-0.037	0.004	0.013
	(0.023)	(0.016)	(0.020)
Urbanicity	-0.011	-0.021	0.016
	(0.020)	(0.014)	(0.017)
Experienced Pandemic Financial Hardship	0.003	0.018	-0.010
	(0.025)	(0.017)	(0.021)
Age Group	0.009	0.009	0.009
	(0.017)	(0.012)	(0.015)
National Identity 1	0.053*	0.028	0.027
	(0.028)	(0.019)	(0.024)
National Identity 2	-0.003	-0.017	-0.019
	(0.025)	(0.017)	(0.021)
Observations	250	256	260
R-squared	0.147	0.139	0.207

Standard errors are given in parenthesis
*p<0.10; **p<0.05; ***p<0.01

Table S13: DE Independent OLS Results

VARIABLES	(1) Mask-wearing	(2) Social-Distancing	(3) Belief in Effectiveness of Measures
Trust in WHO	-0.010	-0.042**	-0.002
	(0.012)	(0.017)	(0.023)
Trust in Science	-0.013	0.013	0.022
	(0.011)	(0.016)	(0.021)
National Institutional Trust	0.013	0.037	0.070**
	(0.019)	(0.027)	(0.035)
National Political Leader Trust	0.031	0.002	-0.003
	(0.021)	(0.030)	(0.039)
Local Institutional Trust	-0.024	0.043*	0.087***
	(0.016)	(0.023)	(0.030)
Trust in Strangers	-0.033***	-0.013	-0.017
	(0.011)	(0.016)	(0.021)

Trust in Employers	0.001	-0.004	0.017
	(0.009)	(0.012)	(0.016)
Local Community Trust	-0.013	0.012	-0.012
	(0.011)	(0.016)	(0.021)
Social Media Trust	-0.020*	0.004	-0.024
	(0.010)	(0.015)	(0.019)
Traditional Media Trust	0.017	-0.013	-0.023
	(0.013)	(0.018)	(0.024)
General Trust (Local)	0.004	-0.029	-0.030
	(0.014)	(0.019)	(0.025)
General Trust (Global)	0.001	0.014	0.029
	(0.014)	(0.019)	(0.025)
Gender (Female)	0.019	0.060**	0.012
	(0.018)	(0.025)	(0.032)
Education Level	0.007	0.011	-0.002
	(0.007)	(0.010)	(0.012)
Medical Experience	0.011	-0.001	-0.008
	(0.009)	(0.013)	(0.016)
Income	0.011	0.010	0.002
	(0.010)	(0.014)	(0.019)
Sufficient Safety Net	0.006	-0.031**	0.001
	(0.010)	(0.014)	(0.018)
# of Household Minors	-0.014	-0.006	-0.012
	(0.016)	(0.023)	(0.030)
Political Ideology (Conservative)	-0.028**	-0.016	0.010
	(0.013)	(0.018)	(0.023)
Religiosity	0.003	0.024	-0.031
	(0.011)	(0.015)	(0.020)
Urbanicity	0.008	-0.030***	-0.022
	(0.008)	(0.011)	(0.015)
Experienced Pandemic Financial Hardship	0.023**	0.006	-0.006
	(0.010)	(0.014)	(0.018)
Age Group	0.000	0.008	-0.010
	(0.010)	(0.014)	(0.018)
National Identity 1	0.026**	-0.001	0.018
	(0.011)	(0.015)	(0.020)
National Identity 2	-0.016	0.001	0.008
	(0.011)	(0.016)	(0.021)
Observations	271	279	282
R-squared	0.224	0.148	0.232

Standard errors are given in parenthesis

*p<0.10; **p<0.05; ***p<0.01

Table S14: ES Independent OLS Results

	(1)	(2)	(3)
VARIABLES	Mask-	Social-	Belief in Effectiveness of

	wearing	Distancing	Measures
Trust in WHO	-0.020	-0.005	0.008
	(0.021)	(0.021)	(0.027)
Trust in Science	0.008	0.032*	0.018
	(0.017)	(0.017)	(0.022)
National Institutional Trust	0.035	0.029	0.053*
	(0.023)	(0.023)	(0.030)
National Political Leader Trust	-0.060**	-0.032	-0.055
	(0.027)	(0.027)	(0.035)
Local Institutional Trust	0.012	0.000	0.029
	(0.015)	(0.015)	(0.020)
Trust in Strangers	-0.044**	-0.033*	-0.092***
	(0.019)	(0.020)	(0.025)
Trust in Employers	0.008	-0.004	0.019
	(0.015)	(0.015)	(0.019)
Local Community Trust	0.009	0.027*	0.032*
	(0.015)	(0.015)	(0.019)
Social Media Trust	0.021	0.032*	-0.025
	(0.018)	(0.018)	(0.024)
Traditional Media Trust	0.020	0.013	-0.011
	(0.017)	(0.017)	(0.022)
General Trust (Local)	0.041*	0.011	0.078***
	(0.021)	(0.021)	(0.027)
General Trust (Global)	-0.043**	-0.010	-0.064**
	(0.019)	(0.019)	(0.025)
Gender (Female)	0.007	-0.025	0.005
	(0.032)	(0.032)	(0.042)
Education Level	0.002	-0.010	0.013
	(0.021)	(0.021)	(0.028)
Medical Experience	0.004	-0.019	0.014
	(0.015)	(0.015)	(0.020)
Income	0.008	0.009	-0.019
	(0.014)	(0.015)	(0.019)
Sufficient Safety Net	0.008	0.008	0.037*
	(0.016)	(0.016)	(0.020)
# of Household Minors	0.001	0.034	-0.004
	(0.024)	(0.024)	(0.031)
Political Ideology (Conservative)	-0.041**	-0.046**	-0.051**
	(0.019)	(0.019)	(0.025)
Religiosity	-0.047**	-0.029	-0.012
	(0.021)	(0.022)	(0.028)
Urbanicity	-0.012	-0.019	0.005
	(0.013)	(0.013)	(0.017)
Experienced Pandemic Financial Hardship	-0.012	0.016	-0.013
	(0.018)	(0.018)	(0.023)
Age Group	0.008	0.041**	0.017
	(0.019)	(0.019)	(0.025)

National Identity 1	0.042***	-0.020	0.004
	(0.015)	(0.015)	(0.019)
National Identity 2	-0.009	0.024	0.019
	(0.019)	(0.019)	(0.024)
Observations	236	234	240
R-squared	0.213	0.220	0.236

Standard errors are given in parenthesis
*p<0.10; **p<0.05; ***p<0.01

Table S15: IL Independent OLS Results

VARIABLES	(1)<br>Mask-wearing	(2)<br>Social-Distancing	(3)<br>Belief in Effectiveness of Measures
Trust in WHO	0.001	0.019	-0.043
	(0.024)	(0.023)	(0.027)
Trust in Science	0.009	-0.033	0.035
	(0.022)	(0.020)	(0.024)
National Institutional Trust	0.032	0.046**	0.042
	(0.024)	(0.022)	(0.027)
National Political Leader Trust	0.010	0.006	-0.028
	(0.030)	(0.029)	(0.034)
Local Institutional Trust	0.031	0.077***	0.054**
	(0.022)	(0.020)	(0.025)
Trust in Strangers	-0.045*	-0.057***	-0.085***
	(0.023)	(0.021)	(0.025)
Trust in Employers	-0.019	-0.027	0.003
	(0.019)	(0.017)	(0.021)
Local Community Trust	-0.002	0.013	-0.009
	(0.018)	(0.016)	(0.020)
Social Media Trust	0.038*	0.008	-0.002
	(0.023)	(0.021)	(0.025)
Traditional Media Trust	-0.020	-0.042*	0.031
	(0.024)	(0.022)	(0.027)
General Trust (Local)	-0.006	0.005	-0.041
	(0.025)	(0.024)	(0.028)
General Trust (Global)	-0.003	0.001	0.062**
	(0.026)	(0.024)	(0.029)
Gender (Female)	0.010	0.067**	-0.066
	(0.036)	(0.034)	(0.040)
Education Level	0.026	0.012	0.022
	(0.019)	(0.018)	(0.021)
Medical Experience	-0.017	-0.012	-0.030
	(0.018)	(0.017)	(0.021)
Income	-0.001	-0.004	-0.010
	(0.019)	(0.018)	(0.022)
Sufficient Safety Net	0.005	0.050***	-0.006
	(0.020)	(0.018)	(0.022)

# of Household Minors	-0.028	-0.001	0.009
	(0.019)	(0.018)	(0.021)
Political Ideology (Conservative)	0.006	0.044**	-0.037
	(0.024)	(0.022)	(0.027)
Religiosity	-0.007	-0.064***	-0.010
	(0.023)	(0.021)	(0.025)
Urbanicity	-0.006	0.011	0.023
	(0.017)	(0.016)	(0.019)
Experienced Pandemic Financial Hardship	-0.038*	0.060***	-0.020
	(0.021)	(0.020)	(0.024)
Age Group	0.035	0.040*	-0.009
	(0.022)	(0.021)	(0.025)
National Identity 1	0.009	-0.044*	0.037
	(0.025)	(0.023)	(0.027)
National Identity 2	0.009	0.028	0.002
	(0.025)	(0.023)	(0.028)
Observations	220	227	228
R-squared	0.156	0.276	0.274

Standard errors are given in parenthesis

*p<0.10; **p<0.05; ***p<0.01

Table S16: AU Independent OLS Results

VARIABLES	(1) Mask-wearing	(2) Social-Distancing	(3) Belief in Effectiveness of Measures
Trust in WHO	-0.001	0.014	-0.005
	(0.030)	(0.018)	(0.021)
Trust in Science	0.091***	0.040**	0.033
	(0.030)	(0.018)	(0.021)
National Institutional Trust	-0.050	0.065**	0.066*
	(0.051)	(0.029)	(0.034)
National Political Leader Trust	0.010	-0.023	-0.009
	(0.049)	(0.030)	(0.035)
Local Institutional Trust	-0.005	0.005	0.043
	(0.042)	(0.025)	(0.029)
Trust in Strangers	-0.071*	-0.058***	-0.059**
	(0.036)	(0.022)	(0.026)
Trust in Employers	0.085***	0.042**	0.021
	(0.030)	(0.018)	(0.021)
Local Community Trust	-0.009	0.001	-0.007
	(0.028)	(0.017)	(0.020)
Social Media Trust	0.022	0.006	0.011
	(0.036)	(0.022)	(0.026)
Traditional Media Trust	-0.019	-0.008	-0.029
	(0.036)	(0.022)	(0.025)
General Trust (Local)	-0.029	-0.015	0.003

	(0.036)	(0.022)	(0.026)
General Trust (Global)	0.025	0.003	-0.006
	(0.033)	(0.020)	(0.024)
Gender (Female)	0.033	0.056*	0.012
	(0.050)	(0.030)	(0.036)
Education Level	0.018	0.021	0.008
	(0.027)	(0.016)	(0.019)
Medical Experience	0.004	0.002	-0.031*
	(0.025)	(0.016)	(0.018)
Income	-0.071**	-0.016	-0.007
	(0.027)	(0.017)	(0.020)
Sufficient Safety Net	0.049	-0.004	0.025
	(0.030)	(0.018)	(0.021)
# of Household Minors	-0.032	0.015	0.001
	(0.033)	(0.019)	(0.023)
Political Ideology (Conservative)	-0.052	0.011	-0.019
	(0.033)	(0.020)	(0.024)
Religiosity	0.041	-0.008	0.027
	(0.027)	(0.017)	(0.020)
Urbanicity	0.018	0.028	-0.009
	(0.031)	(0.018)	(0.022)
Experienced Pandemic Financial Hardship	-0.020	0.009	-0.005
	(0.031)	(0.018)	(0.022)
Age Group	0.023	0.019	0.001
	(0.028)	(0.016)	(0.020)
National Identity 1	0.037	0.004	0.041*
	(0.032)	(0.019)	(0.023)
National Identity 2	-0.021	0.001	-0.025
	(0.038)	(0.022)	(0.027)
Observations	214	243	244
R-squared	0.193	0.203	0.191

Standard errors are given in parenthesis

*p<0.10; **p<0.05; ***p<0.01

Table S17: NZ Independent OLS Results

VARIABLES	(1) Mask-wearing	(2) Social-Distancing	(3) Belief in Effectiveness of Measures
Trust in WHO	-0.023	-0.016	-0.003
	(0.036)	(0.018)	(0.025)
Trust in Science	0.037	0.011	0.055**
	(0.037)	(0.018)	(0.026)
National Institutional Trust	0.176**	0.056	0.049
	(0.071)	(0.036)	(0.050)
National Political Leader Trust	-0.116*	-0.022	-0.013

	(0.068)	(0.035)	(0.048)
Local Institutional Trust	0.004	0.037*	-0.003
	(0.041)	(0.021)	(0.029)
Trust in Strangers	-0.044	0.005	-0.032
	(0.039)	(0.019)	(0.027)
Trust in Employers	0.049	0.002	0.000
	(0.036)	(0.018)	(0.025)
Local Community Trust	0.051	-0.021	0.043
	(0.039)	(0.020)	(0.028)
Social Media Trust	0.063*	0.028	0.008
	(0.036)	(0.018)	(0.024)
Traditional Media Trust	-0.001	-0.021	-0.035
	(0.037)	(0.019)	(0.026)
General Trust (Local)	-0.047	-0.024	-0.001
	(0.048)	(0.023)	(0.032)
General Trust (Global)	-0.047	-0.003	-0.005
	(0.043)	(0.021)	(0.029)
Gender (Female)	0.067	0.001	0.062
	(0.061)	(0.029)	(0.041)
Education Level	0.029	0.009	0.011
	(0.029)	(0.015)	(0.020)
Medical Experience	0.012	0.001	0.000
	(0.029)	(0.015)	(0.021)
Income	0.071**	-0.012	0.033
	(0.031)	(0.016)	(0.022)
Sufficient Safety Net	0.041	0.017	0.011
	(0.031)	(0.015)	(0.021)
# of Household Minors	-0.035	0.012	-0.032
	(0.036)	(0.017)	(0.024)
Political Ideology (Conservative)	-0.011	0.007	-0.029
	(0.037)	(0.018)	(0.025)
Religiosity	-0.028	-0.004	0.010
	(0.032)	(0.016)	(0.023)
Urbanicity	0.059*	-0.013	0.025
	(0.032)	(0.015)	(0.021)
Experienced Pandemic Financial Hardship	0.015	0.017	-0.009
	(0.034)	(0.017)	(0.023)
Age Group	0.016	0.017	-0.035*
	(0.029)	(0.014)	(0.020)
National Identity 1	-0.080*	0.011	0.044
	(0.047)	(0.022)	(0.030)
National Identity 2	0.021	0.017	-0.036
	(0.049)	(0.023)	(0.033)
Observations	173	193	197
R-squared	0.231	0.141	0.193

Standard errors are given in parenthesis

*p<0.10; **p<0.05; ***p<0.01

Table S18: JP Independent OLS Results

VARIABLES	(1)<br>Mask-wearing	(2)<br>Social-Distancing	(3)<br>Belief in Effectiveness of Measures
Trust in WHO	-0.000	0.011	-0.036*
	(0.010)	(0.015)	(0.019)
Trust in Science	-0.002	-0.016	-0.002
	(0.009)	(0.013)	(0.017)
National Institutional Trust	0.045***	0.019	0.035
	(0.014)	(0.021)	(0.028)
National Political Leader Trust	-0.047***	-0.004	-0.004
	(0.016)	(0.024)	(0.031)
Local Institutional Trust	-0.003	0.016	0.002
	(0.012)	(0.018)	(0.023)
Trust in Strangers	-0.028***	-0.035**	-0.049**
	(0.010)	(0.015)	(0.019)
Trust in Employers	0.003	0.026*	0.017
	(0.009)	(0.014)	(0.018)
Local Community Trust	0.007	0.031**	-0.001
	(0.009)	(0.014)	(0.018)
Social Media Trust	-0.014	-0.010	0.015
	(0.011)	(0.016)	(0.021)
Traditional Media Trust	0.005	0.004	0.019
	(0.009)	(0.014)	(0.018)
General Trust (Local)	0.002	0.014	0.039*
	(0.010)	(0.015)	(0.020)
General Trust (Global)	0.007	-0.003	-0.016
	(0.010)	(0.016)	(0.021)
Gender (Female)	0.041***	0.028	0.055*
	(0.015)	(0.022)	(0.029)
Education Level	0.010	0.035**	0.018
	(0.010)	(0.015)	(0.019)
Medical Experience	-0.007	-0.011	-0.003
	(0.009)	(0.014)	(0.018)
Income	-0.001	0.016	0.029
	(0.011)	(0.018)	(0.023)
Sufficient Safety Net	0.005	-0.024*	-0.007
	(0.009)	(0.013)	(0.017)
# of Household Minors	-0.004	-0.017	-0.016
	(0.009)	(0.014)	(0.018)
Political Ideology (Conservative)	0.004	0.001	-0.012
	(0.010)	(0.015)	(0.019)
Religiosity	0.002	-0.000	-0.001
	(0.008)	(0.013)	(0.017)
Urbanicity	0.014*	-0.007	-0.009
	(0.008)	(0.012)	(0.015)
Experienced Pandemic Financial Hardship	0.005	0.026**	0.009

	(0.008)	(0.012)	(0.015)
Age Group	-0.010	0.013	0.014
	(0.008)	(0.012)	(0.015)
National Identity 1	0.014	0.005	-0.002
	(0.009)	(0.014)	(0.018)
National Identity 2	-0.007	-0.003	-0.004
	(0.011)	(0.017)	(0.022)
Observations	307	309	310
R-squared	0.146	0.126	0.085

Standard errors are given in parenthesis
*p<0.10; **p<0.05; ***p<0.01

Table S19: KR Independent OLS Results

VARIABLES	(1)<br>Mask-wearing	(2)<br>Social-Distancing	(3)<br>Belief in Effectiveness of Measures
Trust in WHO	0.035	0.022	-0.074***
	(0.024)	(0.021)	(0.027)
Trust in Science	-0.009	-0.014	0.013
	(0.021)	(0.018)	(0.023)
National Institutional Trust	0.015	0.014	0.009
	(0.028)	(0.025)	(0.033)
National Political Leader Trust	-0.016	0.046*	0.018
	(0.030)	(0.027)	(0.034)
Local Institutional Trust	0.082***	0.055**	0.023
	(0.028)	(0.025)	(0.032)
Trust in Strangers	-0.065***	-0.052***	-0.007
	(0.022)	(0.019)	(0.025)
Trust in Employers	-0.027	0.033*	0.003
	(0.022)	(0.019)	(0.025)
Local Community Trust	0.002	-0.013	0.039
	(0.021)	(0.019)	(0.024)
Social Media Trust	-0.008	-0.012	0.005
	(0.022)	(0.019)	(0.025)
Traditional Media Trust	-0.011	-0.003	-0.030
	(0.025)	(0.022)	(0.029)
General Trust (Local)	-0.008	0.009	0.011
	(0.024)	(0.020)	(0.027)
General Trust (Global)	0.003	-0.009	-0.009
	(0.022)	(0.019)	(0.025)
Gender (Female)	0.051	0.067**	0.028
	(0.032)	(0.028)	(0.036)
Education Level	0.014	-0.003	0.027
	(0.016)	(0.014)	(0.018)
Medical Experience	0.013	0.011	0.019
	(0.013)	(0.012)	(0.015)
Income	0.008	0.003	0.010

	(0.018)	(0.016)	(0.021)
Sufficient Safety Net	0.073***	0.069***	-0.035
	(0.023)	(0.020)	(0.026)
# of Household Minors	-0.020	-0.023	0.000
	(0.017)	(0.015)	(0.019)
Political Ideology (Conservative)	0.031*	0.034**	-0.019
	(0.018)	(0.016)	(0.021)
Religiosity	-0.027*	-0.014	-0.023
	(0.016)	(0.014)	(0.018)
Urbanicity	0.040**	0.023	0.017
	(0.016)	(0.014)	(0.018)
Experienced Pandemic Financial Hardship	-0.011	-0.003	-0.023
	(0.016)	(0.014)	(0.018)
Age Group	0.018	0.031**	-0.014
	(0.016)	(0.014)	(0.018)
National Identity 1	0.014	0.001	0.077***
	(0.024)	(0.021)	(0.028)
National Identity 2	0.064**	0.039	-0.037
	(0.028)	(0.025)	(0.033)
Observations	265	266	282
R-squared	0.313	0.315	0.215

Standard errors are given in parenthesis

*p<0.10; **p<0.05; ***p<0.01

Table S20: TW Independent OLS Results

VARIABLES	(1) Mask-wearing	(2) Social-Distancing	(3) Belief in Effectiveness of Measures
Trust in WHO	0.003	0.006	0.039
	(0.014)	(0.021)	(0.025)
Trust in Science	0.005	0.033*	0.007
	(0.013)	(0.019)	(0.023)
National Institutional Trust	-0.014	-0.005	0.074*
	(0.022)	(0.034)	(0.039)
National Political Leader Trust	0.019	0.007	0.069
	(0.025)	(0.039)	(0.045)
Local Institutional Trust	0.024	0.055*	-0.045
	(0.019)	(0.029)	(0.034)
Trust in Strangers	-0.035***	0.005	-0.015
	(0.013)	(0.019)	(0.023)
Trust in Employers	0.022	-0.001	-0.027
	(0.015)	(0.023)	(0.027)
Local Community Trust	0.029**	0.008	0.001
	(0.012)	(0.018)	(0.022)
Social Media Trust	0.004	0.016	-0.039
	(0.018)	(0.027)	(0.032)

Traditional Media Trust	-0.023	-0.033	-0.001
	(0.016)	(0.025)	(0.029)
General Trust (Local)	-0.012	-0.020	0.029
	(0.017)	(0.026)	(0.031)
General Trust (Global)	0.001	0.025	-0.054*
	(0.016)	(0.024)	(0.029)
Gender (Female)	0.032	0.068**	0.018
	(0.020)	(0.030)	(0.035)
Education Level	0.010	-0.015	0.031
	(0.012)	(0.018)	(0.021)
Medical Experience	-0.016	-0.006	0.025
	(0.020)	(0.032)	(0.036)
Income	0.008	-0.004	0.021
	(0.010)	(0.015)	(0.018)
Sufficient Safety Net	0.011	0.016	-0.027
	(0.013)	(0.019)	(0.022)
# of Household Minors	-0.000	0.023	0.013
	(0.011)	(0.017)	(0.020)
Political Ideology (Conservative)	-0.014	-0.038**	-0.016
	(0.012)	(0.018)	(0.022)
Religiosity	0.004	0.035**	0.006
	(0.011)	(0.017)	(0.020)
Urbanicity	0.006	-0.023	0.041*
	(0.013)	(0.020)	(0.024)
Experienced Pandemic Financial Hardship	0.015	-0.007	0.010
	(0.015)	(0.022)	(0.026)
Age Group	0.011	0.014	-0.006
	(0.011)	(0.017)	(0.019)
National Identity 1	-0.023	-0.042	-0.000
	(0.020)	(0.029)	(0.035)
National Identity 2	0.039	-0.003	0.055
	(0.027)	(0.040)	(0.048)
Observations	222	207	223
R-squared	0.200	0.209	0.240

Standard errors are given in parenthesis
*p<0.10; **p<0.05; ***p<0.01

Table S21: CN Independent OLS Results

VARIABLES	(1) Mask-wearing	(2) Social-Distancing	(3) Belief in Effectiveness of Measures
Trust in WHO	0.010	0.023	-0.006
	(0.017)	(0.017)	(0.023)
Trust in Science	0.010	0.032**	0.020
	(0.016)	(0.016)	(0.022)
National Institutional Trust	-0.035	0.038	0.008

	(0.028)	(0.028)	(0.039)
National Political Leader Trust	0.105***	0.039	-0.040
	(0.032)	(0.032)	(0.044)
Local Institutional Trust	-0.002	0.002	0.066***
	(0.019)	(0.019)	(0.025)
Trust in Strangers	-0.006	-0.010	-0.069***
	(0.012)	(0.011)	(0.016)
Trust in Employers	-0.032**	-0.026*	-0.045**
	(0.015)	(0.015)	(0.020)
Local Community Trust	0.011	0.012	0.012
	(0.013)	(0.013)	(0.018)
Social Media Trust	0.002	0.003	-0.048**
	(0.016)	(0.016)	(0.022)
Traditional Media Trust	-0.017	-0.013	0.011
	(0.019)	(0.018)	(0.025)
General Trust (Local)	-0.008	0.002	-0.020
	(0.019)	(0.019)	(0.026)
General Trust (Global)	0.019	0.004	0.034
	(0.019)	(0.019)	(0.026)
Gender (Female)	0.001	0.043**	0.059**
	(0.021)	(0.020)	(0.028)
Education Level	-0.012	-0.010	-0.010
	(0.014)	(0.014)	(0.020)
Medical Experience	0.005	0.001	0.023*
	(0.009)	(0.009)	(0.012)
Income	-0.011	-0.031***	-0.013
	(0.011)	(0.011)	(0.015)
Sufficient Safety Net	0.041***	0.032**	0.022
	(0.014)	(0.014)	(0.019)
# of Household Minors	-0.016	-0.014	0.039**
	(0.013)	(0.013)	(0.017)
Political Ideology (Conservative)	0.001	0.005	0.007
	(0.011)	(0.011)	(0.015)
Religiosity	0.003	0.023**	0.002
	(0.011)	(0.011)	(0.015)
Urbanicity	0.016	0.024*	0.057***
	(0.014)	(0.014)	(0.019)
Experienced Pandemic Financial Hardship	0.004	-0.008	-0.002
	(0.012)	(0.011)	(0.016)
Age Group	0.008	-0.004	-0.002
	(0.013)	(0.012)	(0.017)
National Identity 1	0.131***	0.086***	0.031
	(0.024)	(0.024)	(0.033)
National Identity 2	-0.005	-0.021	0.057
	(0.027)	(0.027)	(0.038)
Observations	386	376	396
R-squared	0.357	0.361	0.246

Standard errors are given in parenthesis
*p<0.10; **p<0.05; ***p<0.01

Public Health Compliance and Beliefs Per Country/Territory

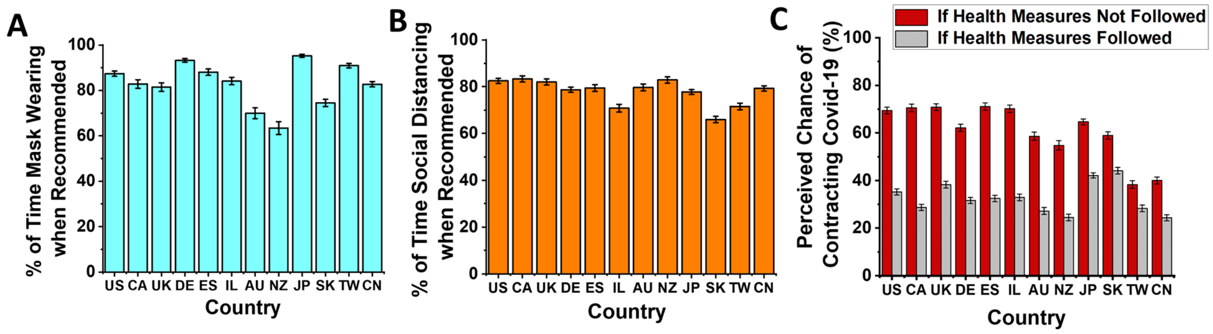

Figure S12: Public health outcomes across countries/territories
Bar plots of the per country/territory mean value of each dependent variable studied in quantitative analysis: (A) The percent of time subjects wear masks when recommended or required to in public spaces, (B) the percent of time subjects social distance when recommended or required to in public spaces, and (C) the percent difference subjects perceive in their chances of contracting Covid-19 if they follow all public health measures reliably minus if no public health measures are followed (belief in effectiveness of measures). Standard ISO 3166 abbreviations for each country/territory are used. Error bars are standard errors.

Standard Deviation Per Regression Variable

Across subjects (*N=3369)* we provide the standard deviation of the regressor variables per country/territory. The three dependent variables are all in percentages units (1-100 in the top three rows of Table S22), while the rest of the variables are on a 1-10 scale. Later, in the regression analyses the percentage units are normalized to 0-1 scale and the independent variables are standardized.

Table S22: Standard deviation (SD) per regression variable across all subjects

	US	CA	UK	DE	ES	IL	AU	NZ	JP	KR	TW	CN	All
Mask-wearing (%)	20.9	29.2	29.5	13.9	21.6	24.3	34.6	37.2	12.6	26.0	14.8	22.9	25.6
Social-distancing (%)	19.2	20.4	20.7	19.0	22.2	25.0	22.8	18.4	18.0	22.8	21.1	22.3	21.6
Belief in Effectiveness (%)	28.9	26.9	26.9	26.1	29.1	30.1	26.9	27.1	22.8	28.5	26.6	29.4	29.2
Local Community Trust	1.79	1.78	1.83	1.34	1.76	1.73	1.59	1.48	1.42	1.58	1.74	1.68	1.68
Local Institutional Trust	1.98	1.66	1.87	1.45	1.98	1.82	1.51	1.74	1.37	1.63	1.60	1.70	1.87
National Institutional Trust	2.16	1.84	2.25	1.79	2.37	2.16	1.89	1.71	1.81	1.87	2.05	1.59	2.20
National Political Leader	2.84	1.95	2.32	1.89	2.31	2.06	2.07	1.88	1.85	1.95	1.92	1.52	2.59
Science Trust	2.00	1.90	1.90	1.78	1.98	1.87	2.02	1.80	1.81	1.86	1.89	1.81	2.01
WHO Trust	2.44	2.26	2.12	2.03	2.26	2.18	2.55	2.31	1.98	2.00	2.09	1.99	2.46
Social Media Trust	2.51	2.18	2.02	1.88	1.88	2.04	2.12	1.94	1.63	2.00	1.83	1.94	2.27
Traditional News Media	2.30	1.96	2.01	1.79	2.16	1.91	2.17	1.93	2.01	1.82	1.86	1.80	2.16
Trust in Employers	2.33	2.12	2.27	1.96	2.20	2.06	2.00	1.91	1.79	1.87	1.84	1.78	2.06
Trust in Strangers	2.14	1.76	1.75	1.53	1.57	1.64	1.70	1.57	1.54	1.75	1.77	2.03	1.82
Transparency: Rationale	2.33	1.73	2.34	1.87	2.36	2.25	2.08	1.93	2.21	2.05	2.15	1.69	2.41
Transparency: Public Feedback	2.51	2.05	2.14	1.96	2.23	2.27	2.19	1.99	1.99	2.00	2.00	1.78	2.41
Transparency: Flexible Guidelines	2.46	1.95	2.31	2.08	2.24	2.28	2.20	2.07	2.00	1.92	2.07	1.76	2.39
Transparency: Officials Educated Enough	2.71	2.26	2.39	2.23	2.48	2.35	2.26	1.84	2.11	2.03	2.10	1.80	2.58
Transparency: Respect Diversity in Communications	2.65	2.03	2.14	1.99	2.27	2.12	2.13	1.91	1.91	1.96	2.52	1.74	2.49
Transparency: Prefer Officials Admit Uncertainty	2.15	1.69	2.17	1.87	2.26	2.35	1.84	1.68	2.01	1.88	2.14	1.82	2.12
National Identity #1	2.10	1.89	2.11	2.25	2.85	2.32	2.61	2.24	2.02	2.21	2.11	1.59	2.26
National Identity #2	2.91	2.35	3.01	2.85	3.08	2.80	2.84	2.65	2.19	2.21	2.07	1.76	2.82
General Trust (local)	1.39	1.17	1.26	1.08	1.24	1.16	1.18	1.14	1.24	1.16	1.16	1.13	1.28
General Trust (global)	1.43	1.17	1.10	1.11	1.30	1.11	1.30	1.19	1.22	1.25	1.17	1.14	1.33

Regression Checks on Transparency SEM Results

Regression checks on transparency SEM results, part 1:

To further shed light on the underlying mechanism that links the institutional trust to the health compliance behavior, we run the following ordinary least squares (OLS) regression format:

$$y_i = \alpha_0 + \beta \cdot Transparency_i + \gamma \cdot X_i + \epsilon_i$$

where y_i stands for the institutional trust. $Transparency_i$ is a multi-dimensional vector of transparency question responses. X_i is a set of control variables for the demographic characteristics as above. Both dependent and independent variables are standardized.

Table S23: Linear regression summary of institutional trust and transparency
(standardized dependent variables)

VARIABLES	(1)<br>Local<br>Institutional Trust	(2)<br>National<br>Institutional Trust	(3)<br>National Political<br>Leader Trust
Transparency: Rationale	0.053	0.172***	0.160***
	(0.041)	(0.043)	(0.043)
Transparency: Public Feedback	0.148***	0.129***	0.148***
	(0.026)	(0.019)	(0.017)
Transparency: Flexible Guidelines	0.130***	0.081**	0.084**
	(0.024)	(0.028)	(0.027)
Transparency: Officials Educated Enough	0.116***	0.222***	0.176***
	(0.034)	(0.026)	(0.028)
Transparency: Respect Diversity in Communications	0.183***	0.082**	0.143***
	(0.022)	(0.032)	(0.037)
Transparency: Prefer Officials Admit Uncertainty	0.063***	0.193***	0.065***
	(0.020)	(0.019)	(0.020)
Gender (Female)	-0.028	-0.014	-0.001
	(0.032)	(0.031)	(0.024)
Education Level	0.034***	0.043***	0.033**
	(0.011)	(0.011)	(0.013)
Medical Experience	0.011	0.018	0.015
	(0.012)	(0.011)	(0.013)
Income	0.002	-0.007	-0.041**
	(0.013)	(0.016)	(0.016)
Sufficient Safety Net	0.109***	0.042**	0.069***
	(0.018)	(0.016)	(0.014)
# of Household Minors	0.007	-0.035**	-0.005
	(0.014)	(0.014)	(0.017)
Political Ideology (Conservative)	-0.044***	-0.089**	0.009
	(0.011)	(0.033)	(0.058)
Religiosity	0.014	-0.004	0.026
	(0.020)	(0.015)	(0.029)
Urbanicity	0.025**	0.008	-0.006
	(0.009)	(0.014)	(0.011)
Experienced Pandemic Financial Hardship	0.034**	-0.022	-0.001
	(0.013)	(0.014)	(0.018)
Age Group	0.009	-0.001	0.014
	(0.022)	(0.014)	(0.015)
Constant	0.141***	-0.144***	-0.372***
	(0.017)	(0.017)	(0.021)
Observations	3,328	3,328	3,328
R-squared	0.460	0.595	0.683

Notes: Standard errors in the parentheses are clustered at country/territory level
*p<0.10; **p<0.05; ***p<0.01

Regression checks on transparency SEM results, part 2:

To verify that the transparency pathway without trust does not improve compliance behavior, as predicted by the SEM analysis, we run the following ordinary least squares (OLS) regression format:

$$y_i = \alpha_0 + \beta \cdot Transparency_i + \gamma \cdot X_i + \epsilon_i$$

where y_i stands for each public health outcome variable. $Transparency_i$ is a multi-dimensional vector of transparency question responses. X_i is a set of control variables for the demographic characteristics as above. Both dependent and independent variables are standardized.

Table S24: Linear regression summary of transparency and public health behavior
(standardized variables)

VARIABLES	(1) Mask-Wearing	(2) Social-Distancing	(3) Belief in Effectiveness of Measures
Transparency: Rationale	0.014***	0.001	0.015*
	(0.004)	(0.006)	(0.008)
Transparency: Public Feedback	-0.002	-0.001	0.001
	(0.007)	(0.007)	(0.007)
Transparency: Flexible Guidelines	-0.000	0.021***	0.018**
	(0.009)	(0.006)	(0.008)
Transparency: Officials Educated Enough	0.012	0.003	-0.000
	(0.010)	(0.008)	(0.006)
Transparency: Respect Diversity in Communications	-0.015*	-0.006	-0.014
	(0.007)	(0.006)	(0.014)
Transparency: Prefer Officials Admit Uncertainty	0.016**	0.021***	0.020*
	(0.006)	(0.004)	(0.009)
Gender (Female)	0.027***	0.040***	0.027**
	(0.009)	(0.010)	(0.012)
Education Level	0.008*	0.007	0.010
	(0.005)	(0.006)	(0.006)
Medical Experience	0.008**	0.004	0.004
	(0.003)	(0.003)	(0.009)
Income	-0.000	-0.009	0.005
	(0.007)	(0.005)	(0.005)
Sufficient Safety Net	0.019***	0.014*	0.004
	(0.006)	(0.007)	(0.007)
# of Household Minors	-0.014***	0.002	-0.009
	(0.004)	(0.006)	(0.007)

Political Ideology (Conservative)	-0.020***	-0.010*	-0.033***
	(0.005)	(0.005)	(0.006)
Religiosity	-0.012*	-0.004	-0.015*
	(0.006)	(0.006)	(0.007)
Urbanicity	0.015**	-0.002	0.014**
	(0.006)	(0.008)	(0.006)
Experienced Pandemic Financial Hardship	-0.004	0.007	-0.022***
	(0.005)	(0.006)	(0.006)
Age Group	0.006	0.020***	-0.009
	(0.004)	(0.003)	(0.007)
Constant	0.869***	0.810***	0.340***
	(0.006)	(0.007)	(0.006)
Observations	3,113	3,155	3,240
R-squared	0.149	0.098	0.161

Notes: Standard errors in the parentheses are clustered at country/territory level
*p<0.10; **p<0.05; ***p<0.01

Information Trust and Public Health Compliance and Beliefs

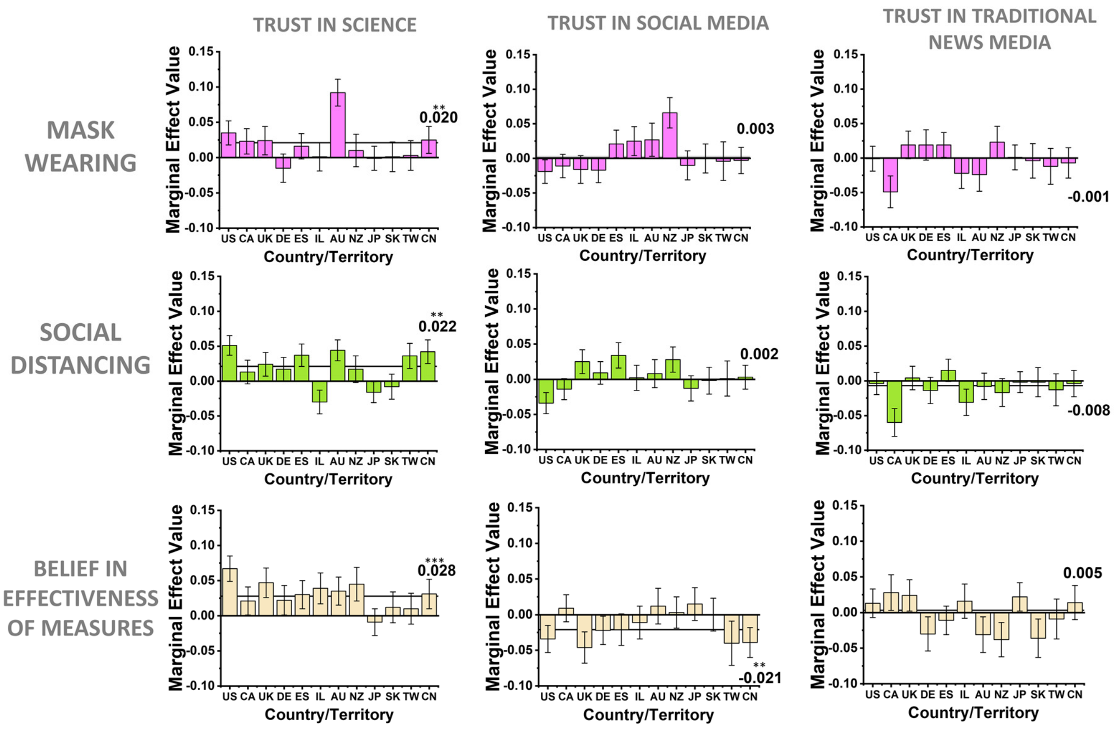

Figure S13: Per country/territory marginal effects for which type of information pathway trust improves public health compliance and beliefs

Heterogenous per country/territory marginal effects for the information-related trust categories of trust in science, trust in social media, and trust in traditional news media, for each public health

outcome variable: mask wearing, social distancing, and belief in the effectiveness of public health measures. Error bars are standard errors of the mean, and the global coefficient value from Table 1 is marked with the horizontal line on each plot (*p<0.10; **p<.05; ***p<.01). Independent variables only are standardized, dependent variables are kept as percentages (0-1 scale).

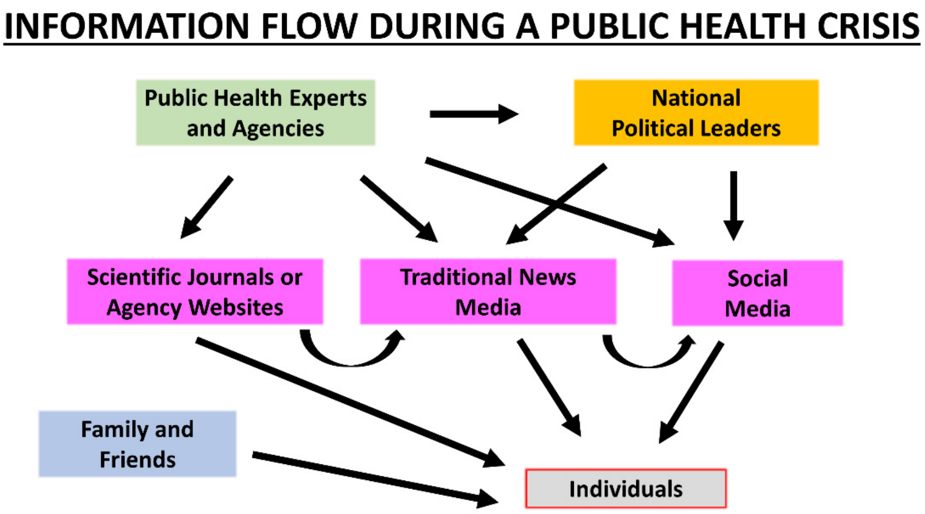

Figure S14: Pathways of information flow during a public health crisis
While medical professionals and public health-related researchers tend to be the original source of most expert pandemic information, the information itself often passes through multiple entities before reaching the public. This "telephone game" of information flow risks distortion or loss of scientific information during public health crises. Thus, partly, the strong positive effects from scientifically-oriented national institutions on public health behaviors and beliefs we observed in our report may result from the recognition by the public that these institutions are trustworthy primary information sources.

Community-related Trust and Public Health Compliance and Support

Extended version of main text "Trust in Local Communities & General Trust" section:
Putnam, Fukuyama, Yamagishi, and others have written extensively on the importance of trust in local communities and general trust for achieving a variety of different large scale cooperative enterprises within societies (1, 15–17). We verified this concept in the pandemic context, finding in regression results that higher trust in friends and family (local community trust) was correlated with higher compliance and support for public health measures (Table 1, Fig. S15). In addition, SEM analysis revealed that local community trust led to higher public health compliance only when it strengthens local institutional trust (Fig. S16). These results agreed generally with prior community-focused trust research (15, 16). However, our results showed that, while local community trust was predictive of public health compliance, Yamagishi's general trust (1) was not a significant predictor (Table 1). On the other hand, lower trust in strangers and acquaintances was found to correlate with higher public health compliance and belief in

effectiveness of measures (Table 1, Fig. S15). This last finding agrees with a prior report, where lower social trust was found to correlate with stronger identification of strangers as infection threats (18).

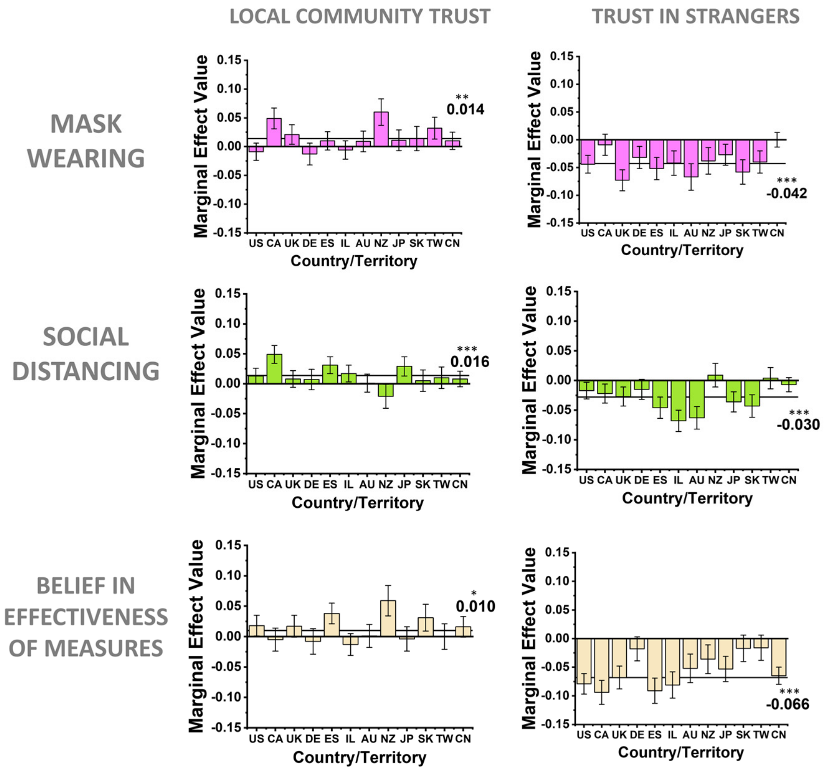

Figure S15: Per country/territory marginal effects for how community-related trust improves public health compliance and beliefs
Heterogenous per country/territory marginal effects for the community-related trust categories of trust in local communities (friends and family) and trust in strangers (strangers and visiting local businesses), for each public health outcome variable: mask wearing, social distancing, and belief in the effectiveness of public health measures. Error bars are standard errors, and the global coefficient value from Table 1 is marked with the horizontal line on each plot (*p<0.10; **p<0.05; ***p<.01). Independent variables only are standardized, dependent variables are kept as percentages (0-1 scale).

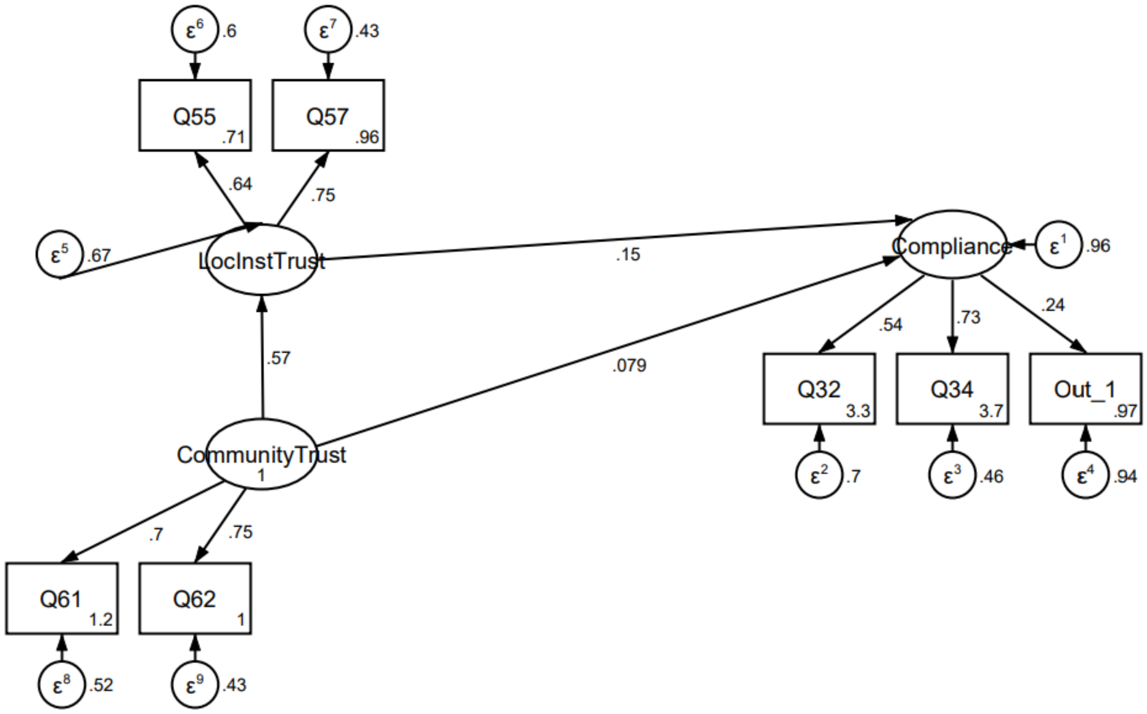

Figure S16: Structural equation modeling (SEM) for local community trust, public health compliance & beliefs, and local institutional trust
Screenshot of SEM model built in STATA with standardized coefficients reported, for testing the relationship between local community trust ("CommunityTrust"), local institutional trust ("LocInstTrust"), and public health behaviors ("Compliance"). Question numbers match the survey questions, other than "Out_1" which is Q42-Q43. Standardized coefficients are reported above each pathway. RMSEA for this model is 0.033. Additional model information from STATA output is provided in Supplementary Appendix 4. This model was tested conceptually based on prior theoretical work (15, 16) and the moderate correlation observed between local community trust and local institutional trust (r = 0.40). In contrast, community trust was uncorrelated with national political leader trust (r = 0.24) and national institutional trust (r = 0.22).

General Trust (Yamagishi Scale)

Yamagishi's general trust questions (11) (though refined into in-group and out-group formulations (19)) were used to construct this general trust index used in the Table 1 regression. Yamagishi's general trust score (the mean of six questions) was calculated based on the local community scale (Q40, Q65-69) and "global" scale (Q40, Q88-92). These measures were found to not be significant in predicting public health compliance or support (Table 1).

National Identity

For psychological factors, only one national identity question from Van Bavel et al. was found to be a significant predictor of public health behaviors in our results: "I identify as an (American, Canadian, etc.)" was positively significant in some outcome variables (Table 1), somewhat agreeing with prior results (20). Unfortunately, national identity is difficult to interpret, or to use

to plan policy, since the question is so general and vague, and may be interpreted quite variably across cultures and individuals.

Variance Inflation Factor Multicollinearity Test

Table S25: Variance Inflation Factor (VIF) Test For Table 1 Regressors

Variable	VIF	1/VIF
Trust in WHO	1.83	0.547
Trust in Science	1.70	0.586
National Institutional Trust	3.37	0.296
National Political Leader Trust	3.82	0.261
Local Institutional Trust	2.22	0.450
Trust in Strangers	1.62	0.619
Trust in Employers	1.46	0.682
Local Community Trust	1.43	0.701
Social Media Trust	1.91	0.524
Traditional Media Trust	2.06	0.484
General Trust (Local)	2.43	0.410
General Trust (Global)	2.41	0.415
Gender (Female)	1.08	0.928
Education Level	1.17	0.852
Medical Experience	1.14	0.878
Income	1.36	0.736
Sufficient Safety Net	1.41	0.707
# of Household Minors	1.58	0.634
Political Ideology	1.34	0.745
Religiosity	1.28	0.779
Urbanicity	1.20	0.835
Financial Hardship	1.33	0.752
Age Group	1.20	0.832
National Identity 1	2.08	0.481
National Identity 2	2.48	0.402
Country/Territory Dummy Variables (US is base country)		
UK	1.81	0.553
CA	1.81	0.552
DE	2.11	0.473
ES	1.90	0.525
IL	1.74	0.573
AU	1.77	0.564
NZ	1.84	0.544
JP	2.17	0.460
KR	2.11	0.474
TW	2.01	0.497
CN	2.85	0.350
**Mean VIF**	**1.86**	

Major Trends in Demographics Per Country/Territory Summary

Overview:
As summarized from Table 1, the following statistically significant demographic trends were noted:

- Age increase was correlated with greater compliance with measures, but did not correlate with increased belief in effectiveness of measures
- Being female was correlated both with greater compliance and belief in the effectiveness of measures.
- Being more educated was correlated with increased mask wearing only.
- Being politically conservative was correlated with reduced mask wearing and decreased belief in effectiveness of measures.
- Experiencing financial hardship was correlated with decreased belief in effectiveness of measures only, not behavioral compliance.
- Living in a city was correlated with increased belief in effectiveness of measures and mask wearing.
- Prior medical experience was correlated with higher mask wearing frequency only.
- Having a sufficient safety net in life generally was correlated with higher mask wearing.
- Having more kids was correlated with worse mask wearing.
- Higher income was correlated with worse social distancing only.
- Religiosity did not have significant effects on any outcome variable.

Per Country/Territory Demographics Tables:

See Supplementary Data for more complete demographic information, this is just a brief summary as a guide. The *N* values are the final subjects after examining three attention check short answer questions for baseline understanding and effort of the survey questions. For Prolific and Crowdworks more than ~95% of subjects passed the attention checks (e.g. showing on at least one short answer response check that the meaning of the survey was understood). For QuestionPro, the survey company only kept responses that passed attention checks.

Table S26: United States of America, Core Demographics

Variable	Statistics (N= 350 before, N=344 after removing duplicates)
Gender	56.1% Male, 43.0% Female, rest Other
Age	34.9% 18-29, 50.0% 30-45, 13.3% 46-59, rest 60+
Race (multiple selections allowed)	Caucasian 74.9%, Black or African Descent 11.4%, Hispanic or Latino/Latinx 8%, East Asian 6.9%, South Asian or Pacific Islander 3.7%, Middle Eastern 0.9%, rest Other
Education (earned or in progress)	20.9% High School Degree, 45.3% Bachelor's Degree, 26.2% Master's Degree, 7.6% Beyond Master's Degree
Salary (USD)	10.8% < 20,000, 20.6% 20-50,000, 35.2% 50-100,000, 26.6% 100-200,000, rest 100-200,000 or no income
Political Preference (scale 1-10, liberal to conservative)	Mean 4.41, Median 4.00
Religiousness Preference (scale 1-10, not religious to very religious)	Mean 4.62, Median 4.00

See Supplementary Data for full data. True school life expectancy in the United States is 16 years. True ethnicity is White 72.4%, Hispanic 16.3%, Black 12.6%, Asian 4.8%, Amerindian and Alaska Native 0.9%, Native Hawaiian and other Pacific Islander 0.2%, other 6.2%, two or more races 2.9% (2010 est.) (CIA World Factbook).

Table S27: Canada, Core Demographics

Variable	Statistics (N=256 before, N=254 after removing duplicates)
Gender	53.1% Male, 46.5% Female, rest Other
Age	44.8% 18-29, 45.7% 30-45, 6.7% 46-59, rest 60+
Race (multiple selections allowed)	Caucasian 60.9%, Black or African Descent 9%, Hispanic or Latino/Latinx 1.6%, East Asian 18.4%, South Asian or Pacific Islander 9%, Middle Eastern 0.8%, rest Other
Education (earned or in progress)	12.1% High School Degree, 62.5% Bachelor's Degree, 18% Master's Degree, rest beyond Master's Degree or no High School Degree
Salary (Canadian Dollars)	10.5% < 20,000, 21.5% 20-50,000, 39.1% 50-100,000, 21.1% 100-200,000, rest >200,000 or no income
Political Preference (scale 1-10, liberal to conservative)	Mean 4.02, Median 4.00
Religiousness Preference (scale 1-10, not religious to very religious)	Mean 3.91, Median 3.00

See Supplementary Data for full data. True school life expectancy in Canada 16 is years (CIA World Factbook). The true ethnicity distribution is Canadian 32.3%, English 18.3%, Scottish 13.9%, French 13.6%, Irish 13.4%, German 9.6%, Chinese 5.1%, Italian 4.6%, North American Indian 4.4%, East Indian 4%, other 51.6% (2016 est.) (CIA World Factbook).

Table S28: United Kingdom, Core Demographics

Variable	Statistics (N=273, no cut)
Gender	42.9% Male, 57.1% female
Age	29.3% 18-29, 41% 30-45, 19.8% 46-59, 9.9% 60+
Race (multiple selections allowed)	Caucasian 91.6%, Black or African Descent 2.2%, East Asian 1.8%, rest Other
Education (earned or in progress)	26.7% High School Degree, 48% Bachelor's Degree, 13.9% Master's Degree, rest beyond Master's Degree or no High School Degree
Salary (British Pounds)	19.8% < 20,000, 48% 20-50,000, 26.4% 50-100,000, rest 100-200,000 or no income
Political Preference (scale 1-10, liberal to conservative)	Mean 4.44, Median 4.00
Religiousness Preference (scale 1-10, not religious to very religious)	Mean 2.9, Median 2.00

See Supplementary Data for full data. True school life expectancy in the United Kingdom is 17 years (CIA World Factbook) (2018).

Table S29: Germany, Core Demographics

Variable	Statistics (N=290 before, N=289 after removing duplicates)
Gender	63.1% Male, 35.2% Female
Age	59.7% 18-29, 33.8% 30-45, rest 46-59 or 60+
Race (multiple selections allowed)	Caucasian 85.5%, Middle Eastern 4.8%, Southeast Asian and Pacific Islanders 2.4%, East Asian 1.7%, rest Other
Education (earned or in progress)	11.7% No High School Diploma, 25.2% High School Diploma, 36.6% Bachelor's Degree, 20% Master's Degree, rest beyond Master's Degree
Salary (Euros)	10% no income, 26.9% < 17,000, 32.4% 17-42,000, 21.7% 42-85,000, 85-170,0000 8.3%, rest >170,000
Political Preference (scale 1-10, liberal to conservative)	Mean 3.82, Median 4.00
Religiousness Preference (scale 1-10, not religious to very religious)	Mean 2.62, Median 2.00

See Supplementary Data for full data. True school life expectancy in Germany is 17 (CIA World Factbook) (2018). The true ethnicity distribution is German 86.3%, Turkish 1.8%, Polish 1%, Syrian 1%, Romanian 1%, other/stateless/unspecified 8.9% (CIA World Factbook) (2020 est.).

Table S30: Spain, Core Demographics

Variable	Statistics (N=253, no cut)
Gender	69.3% Male, 29.1% Female
Age	67.3% 18-29, 25.2% 30-45, rest 46-59 or 60+
Race (multiple selections allowed)	94.5% Spanish, rest Other
Education (earned or in progress)	19.3% Bachillerato, 55.9% Diplomatura/licenciatura/etc., 14.6% Master, rest Other (see supplementary data)
Salary (Euros)	23.6% < 15,000, 30.3% 15-25,000, 20.1% 25-35,000, 8.7% 35-40,000, 11.8% >40,000, rest no income
Political Preference (scale 1-10, liberal to conservative)	Mean 3.73, Median 3.00
Religiousness Preference (scale 1-10, not religious to very religious)	Mean 2.34, Median 1.00

See Supplementary Data for full data. True school life expectancy in Spain is 18 years (2018) (CIA World Factbook). The true ethnicity distribution is Spanish 84.8%, Moroccan 1.7%, Romanian 1.2%, other 12.3% (2021 est.) (CIA World Factbook).

Table S31: Israel, Core Demographics

Variable	Statistics (N=243 before cuts, N=234 after removing duplicates)
Gender	44.9% Male, 54.7% Female, rest Other
Age	68.8% 18-29, 23.1% 30-45, 6.8% 46-59, rest 60+
Race (multiple selections allowed)	88.0% Jewish, 6.0% non-Jewish Arab, rest Other
Education (earned or in progress)	26.5% High School Degree, 50.4% Bachelor's Degree, 17.1% Master's Degree, rest beyond Master's Degree or no High School Degree
Salary (Israeli Shekel)	32.1% < 75,000, 27.4% 75-150,000, 18.4% 150-300,000, 8.6% 300-600,000, 12.4% no income, rest >600,000
Political Preference (scale 1-10, liberal to conservative)	Mean 4.28, Median 4.00
Religiousness Preference (scale 1-10, not religious to very religious)	Mean 3.47, Median 2.00

See Supplementary Data for full data. True school life expectancy in Israel is 16 years (2018) (CIA World Factbook). The true ethnicity distribution is Jewish 74.1% (of which Israel-born 78.1%, Europe/America/Oceania-born 15.2%, Africa-born 4.3%, Asia-born 2.4%), Arab 21%, other 4.9% (2019 est.) (CIA World Factbook).

Table S32: Australia, Core Demographics

Variable	Statistics (N=254, N=253 after removing 1 NaN entry)
Gender	56.9% Male, 41.9% Female, rest Other
Age	53.7% 18-29, 37.7% 30-45, rest 46-59 or 60+
Race (multiple selections allowed)	47.1% English, 16% Chinese, 16.3% Native Australian, rest mixed European descents or Other
Education (earned or in progress)	18.3% High School Degree, 56.4% Bachelor's Degree, 18.7% Master's Degree, rest beyond Master's Degree or no High School Degree
Salary (Australian Dollars)	8.6% < 18,000, 9.3% 18-37,000, 40.5% 37-90,000, 31.5% 90-180,000, rest >180,000 or no income
Political Preference (scale 1-10, liberal to conservative)	Mean 4.1, Median 4.00
Religiousness Preference (scale 1-10, not religious to very religious)	Mean 3.43, Median 2.00

See Supplementary Data for full data. True school life expectancy in Australia is 21 (CIA World Factbook) (2018). The true ethnicity distribution is English 25.9%, Australian 25.4%, Irish 7.5%, Scottish 6.4%, Italian 3.3%, German 3.2%, Chinese 3.1%, Indian 1.4%, Greek 1.4%, Dutch 1.2%, other 15.8% (includes Australian Aboriginal .5%), unspecified 5.4% (CIA World Factbook) (2011 est.).

Table S33: New Zealand, Core Demographics

Variable	Statistics (N=207, no cut)
Gender	51.9% Male, 47.1% Female, rest Other
Age	39.5% 18-29, 42.9% 30-45, 12.4% 46-59, or rest 60+
Race (multiple selections allowed)	68.6% European Descent, 25.7% Asian Descent, 4.8% Indigenous/Maori, rest Other
Education (earned or in progress)	23.3% High School Degree, 54.3% Bachelor's Degree, 15.7% Master's Degree, rest beyond Master's Degree or no High School Degree
Salary (New Zealand Dollars)	10% < 14,000, 20.5% 14-48,000, 21.4% 48-70,000, 37.6% 70-150,000, 7.6% 150,000, rest no income
Political Preference (scale 1-10, liberal to conservative)	Mean 3.66, Median 3.00
Religiousness Preference (scale 1-10, not religious to very religious)	Mean 3.17, Median 2.00

See Supplementary Data for full data. True school life expectancy in New Zealand is 19 years (CIA World Factbook) (2018). The true ethnicity distribution is European 64.1%, Maori 16.5%, Chinese 4.9%, Indian 4.7%, Samoan 3.9%, Tongan 1.8%, Cook Islands Maori 1.7%, English 1.5%, Filipino 1.5%, New Zealander 1%, other 13.7% (2018 est.) (CIA World Factbook).

Table S34: Japan, Core Demographics

Variable	Statistics (N=327 before, N=318 after removing duplicates)
Gender	46.5% Male, 52.5% Female, rest Other
Age	15.4% 18-29, 56.6% 30-45, 23.9% 46-59, rest 60+
Race (multiple selections allowed)	99.7% Japanese East Asian, rest Other
Education (earned or in progress)	22.6% High School Only, 65.1% Bachelor's, rest Other (see Japan's survey form)
Salary (10,000 Japanese yen)	13.8% < 200, 46.9% 20-500, 33.6% 500-1000, rest no income or above 1000
Political Preference (scale 1-10, liberal to conservative)	Mean 5.98, Median 6.00
Religiousness Preference (scale 1-10, not religious to very religious)	Mean 3.36, Median 3.00

See Supplementary Data for full data. True school life expectancy in Japan is 15 years (CIA World Factbook) (2016). The true ethnicity distribution is Japanese 97.9%, Chinese 0.6%, Korean 0.4%, other 1.1% (includes Vietnamese, Filipino, and Brazilian) (2017 est.) (CIA World Factbook).

Table S35: Taiwan, Core Demographics

Variable	Statistics (N=254 before, N=239, after removing 14 duplicates)
Gender	47.7% Male, 52.3% Female
Age	25.5% 20-29, 47.7% 30-45, 20.9% 46-59, rest 60+
Race (multiple selections allowed)	96.8% East Asian, rest Other
Education (earned or in progress)	14.2% High School Only, 51.0% Bachelor's, 28.0% Master's, rest below High School or Other
Salary (Taiwanese Dollars)	8.4% < 30,000, 36.4% 30-50,000, 15.5% 50-70,000, 15.5% 70-100,000, 10.5% 10-160,000, (18 subjects wrote prefer not to answer, this option was only given in Taiwan and was later set to NaN for the analysis), rest no income
Political Preference (scale 1-10, liberal to conservative)	Mean 4.91, Median 5.00
Religiousness Preference (scale 1-10, not religious to very religious)	Mean 5.68, Median 6.00

See Supplementary Data for full data. The true ethnicity distribution in Taiwan is Han Chinese (including Holo, who compose approximately 70% of Taiwan's population, Hakka, and other groups originating in mainland China) more than 95%, indigenous Malayo-Polynesian peoples 2.3% (CIA World Factbook).

Table S36: South Korea, Core Demographics

Variable	Statistics (N=299, no cut)
Gender	43.1% Male, 55.9% Female, rest Other
Age	48.5% 18-29, 36.8% 30-45, 12.4% 46-59, rest 60+
Race (multiple selections allowed)	95.4% East Asian, rest Other
Education (earned or in progress)	25.4% High School, 52% Bachelor's, 14.1% Master's, 4.7% beyond Master's, rest below High School
Salary (Korean won)	16.72% < 20,000, 36.8% 20-50,000, 27.0% 50-100,000, 9.0% 100-200,000, rest no income or above 200,000
Political Preference (scale 1-10, liberal to conservative)	Mean 4.9, Median 5.00
Religiousness Preference (scale 1-10, not religious to very religious)	Mean 3.91, Median 4.00

See Supplementary Data for full data. True school life expectancy in South Korea is 17 (2018) (CIA World Factbook). The true ethnicity distribution is "homogeneous" (CIA World Factbook).

Table S37: China, Core Demographics

Variable	Statistics (N=406, no cut)
Gender	48.8% Male, 51.2% Female
Age	43.8% 18-29, 47.8% 30-45, 7.9% 46-59, rest 60+
Race (multiple selections allowed)	94.6% East Asian, rest Other
Education (earned or in progress)	7.9% High School, 76.9% Bachelor's, 11.1% Master's, 2.5% beyond Master's, rest below High School
Salary (RMB)	18.0% < 131,662, 37.2% 131,662-329,155, 20.9% 329,155-658,310, 13.1% 658,310-1,316,620, 8.4% above 1,316,620, rest no income
Political Preference (scale 1-10, liberal to conservative)	Mean 4.12, Median 4.00
Religiousness Preference (scale 1-10, not religious to very religious)	Mean 3.75, Median 2.00

See Supplementary Data for full data. The true ethnicity distribution is Han Chinese 91.6%, Zhuang 1.3%, other (includes Hui, Manchu, Uighur, Miao, Yi, Tujia, Tibetan, Mongol, Dong, Buyei, Yao, Bai, Korean, Hani, Li, Kazakh, Dai, and other nationalities) 7.1% (2010 est.) (CIA World Factbook).

Supplementary Information References

1. Yamagishi, T. *Trust: The Evolutionary Game of Mind and Society*; The Science of the Mind; Springer: Tokyo, Japan, 2011; ISBN 978-4-431-53935-3.
2. Li, L. Political Trust in Rural China. *Mod. China* **2004**, *30,* 228–258. https://doi.org/10.1177/0097700403261824.
3. Baniamin HM, Jamil I, Askvik S. Mismatch between lower performance and higher trust in the civil service: Can culture provide an explanation? International Political Science Review. **2020**,41,192-206. doi:10.1177/0192512118799756
4. Ma, D., Yang, F. Authoritarian Orientations and Political Trust in East Asian Societies.*East Asia*,**2014**, *31*,323–341. https://doi.org/10.1007/s12140-014-9217-z
5. Q. Yang, W. Tang, Exploring the Sources of Institutional Trust in China: Culture, Mobilization, or Performance?: Exploring the Sources of Institutional Trust in China. *Asian Politics & Policy,* **2010**,*2*,415–436
6. Liu J, Zhang L, Yan Y, Zhou Y, Yin P, Qi J, Wang L, Pan J, You J, Yang J et al. Excess mortality in Wuhan city and other parts of China during the three months of the covid-19 outbreak: findings from nationwide mortality registries. *BMJ*. **2021**, *372:n415*. doi: 10.1136/bmj.n415.
7. Karlinsky, A., & Kobak, D. (2021). The World Mortality Dataset: Tracking excess mortality across countries during the COVID-19 pandemic. *medRxiv : the preprint server for health sciences*, **2021**, *preprint*.
8. Cook, K.S.; Hardin, R.; Levi, M. *Cooperation without Trust*? Russell Sage Foundation: Manhattan, NY, USA, 2005; ISBN 978-1-61044-135-3.
9. Mann, M.; Schleifer, C. Love the Science, Hate the Scientists: Conservative Identity Protects Belief in Science and Undermines Trust in Scientists. *Soc. Forces* **2020**, *99*, 305–332. https://doi.org/10.1093/sf/soz156.
10. Inoguchi, T., Tokuda, Y. *Trust with Asian Characteristics: Interpersonal and Institutional*;; Springer: Singapore, 2017; ISBN 978-981-10-2304-0.
11. T. Yamagishi, M. Yamagishi, Trust and commitment in the United States and Japan. *Motiv Emot*, **1994**,*18*, 129–166.
12. A. Bell, M. Fairbrother, K. Jones, Fixed and random effects models: making an informed choice. *Qual Quant*, **2019**,*53*, 1051–1074.
13. P. Suthaharan, *et al.*, Paranoia and belief updating during the COVID-19 crisis. *Nat Hum Behav*, **2021**, *59*,1–13
14. Bryan, M.L.; Jenkins, S.P. Multilevel Modelling of Country Effects: A Cautionary Tale. *Eur. Sociol. Rev.* **2016**, *32*, 3–22 .
15. Putnam, R. The Prosperous Community: Social Capital and Public Life. *Am. Prospect.* **1993**, *4*, 35–42.
16. Fukuyama, F. *Trust: The social virtues and the creation of prosperity*; 1. Free Press paperback ed.; Free Press: New York, NY, USA, 1996; ISBN 978-0-684-82525-0.
17. Putnam, R.D. *Bowling Alone: The Collapse and Revival of American Community*; Simon and Schuster: Cammeray, Australia, 2000; ISBN 978-0-7432-0304-3.
18. A. Olivera-La Rosa, E. G. Chuquichambi, G. P. D. Ingram, Keep your (social) distance: Pathogen concerns and social perception in the time of COVID-19. *Pers Individ Dif* **166**, 110200 (2020).
19. Sturgis, P., & Smith, P. Assessing the validity of generalized trust questions: What kind of trust are we measuring? International Journal of Public Opinion Research, 2010,22, 74–92
20. Van Bavel, J.J.; Cichocka, A.; Capraro, V.; Sjåstad, H.; Nezlek, J.B.; Pavlović, T.; Alfano, M.; Gelfand, M.J.; Azevedo, F.; Birtel, M.D.; et al. National Identity Predicts Public Health Support during a Global Pandemic. *Nat. Commun.* **2022**, *13*, 517. https://doi.org/10.1038/s41467-021-27668-9.
